# Hem-1 regulates protective humoral immunity and limits autoantibody production in a B cell–specific manner

**DOI:** 10.1172/jci.insight.153597

**Published:** 2022-05-09

**Authors:** Alan Avalos, Jacob T. Tietsort, Nutthakarn Suwankitwat, Jonathan D. Woods, Shaun W. Jackson, Alexandra Christodoulou, Christopher Morrill, H. Denny Liggitt, Chengsong Zhu, Quan-Zhen Li, Kevin K. Bui, Heon Park, Brian M. Iritani

**Affiliations:** 1 The Department of Comparative Medicine, University of Washington, Seattle, Washington, USA.; 2 Seattle Children’s Research Institute, Seattle, Washington, USA.; 3 Department of Immunology, Microarray and Immune Phenotyping Core Facility, University of Texas Southwestern Medical Center, Dallas, Texas, USA.

**Keywords:** Immunology, Autoimmune diseases, Bacterial infections, Cytoskeleton

## Abstract

Hematopoietic protein-1 (Hem-1) is a member of the actin-regulatory WASp family verprolin homolog (WAVE) complex. Loss-of-function variants in the *NCKAP1L* gene encoding Hem-1 were recently discovered to result in primary immunodeficiency disease (PID) in children, characterized by poor specific Ab responses, increased autoantibodies, and high mortality. However, the mechanisms of how Hem-1 deficiency results in PID are unclear. In this study, we utilized constitutive and B cell–specific *Nckap1l*-KO mice to dissect the importance of Hem-1 in B cell development and functions. B cell–specific disruption of Hem-1 resulted in reduced numbers of recirculating follicular (FO), marginal zone (MZ), and B1 B cells. B cell migration in response to CXCL12 and -13 were reduced. T-independent Ab responses were nearly abolished, resulting in failed protective immunity to *Streptococcus* *pneumoniae* challenge. In contrast, T-dependent IgM and IgG2c, memory B cell, and plasma cell responses were more robust relative to WT control mice. B cell–specific Hem-1–deficient mice had increased autoantibodies against multiple autoantigens, and this correlated with hyperresponsive BCR signaling and increased representation of CD11c^+^T-bet^+^ age-associated B cell (ABC cells) — alterations associated with autoimmune diseases. These results suggest that dysfunctional B cells may be part of a mechanism explaining why loss-of-function Hem-1 variants result in recurring infections and autoimmunity.

## Introduction

B cells constitute a portion of the adaptive immune system responsible for producing protective antibodies that mark pathogens and malignant cells for destruction. These functions are mediated, in part, through recognition of specific antigens by the B cell receptor (BCR) complex, which — upon antigen binding and interaction with coreceptors — activates signal transduction cascades, resulting in B cell proliferation and maturation into memory B cells or Ab-producing plasma cells (see ref. 1 for review). Ab responses following B cell activation can be further divided to long-lived, highly specific T-dependent (TD) responses and rapidly generated but shorter lived, T-independent (TI) responses. TI Ab responses, which consist mostly of the IgM isotype, are initiated in response to TI antigens such as bacterial polysaccharides, LPS, and flagella, which are capable of crosslinking BCRs and TLRs on innate-like marginal zone (MZ) and B1 B cells. In contrast, TD responses require mature follicular (FO) B cells to interact with T follicular helper (Tfh) cells in germinal center (GC) reactions, which provide costimulatory signals and T cell–derived cytokines, leading to memory B cell and plasma cell formation. Upon Ag reencounter, memory B cells differentiate into plasmablasts and plasma cells capable of producing class-switched Abs such as IgG1, IgG2a/c, IgG3, IgA, and IgE.

The recent use of advanced imaging techniques has revealed that dynamic aspects of BCR signaling and responses are regulated, in part, by reorganization of the actin cytoskeleton (see refs. 2–4 for review). These dynamic aspects of BCR signaling and responses include receptor clustering and formation of the immunological synapse, recruitment and activation of signaling molecules, cytokine release, B cell adhesion and migration, and chromatin modifications controlling transcription. Actin polymerization is induced by BCR-ligand interaction, which leads to activation of Guanine nucleotide exchange factors (GEFs) such as Vav, Dock2, and Dock8, which then activate the Rho family of GTPases including Rac1/2 and Cdc42 (5, 6). GTP-bound Cdc42 then interacts and activates the Wiskott-Aldrich Syndrome protein (WASp) regulatory complex**,** whereas GTP-Rac predominantly activates the WASp family verprolin homolog (WAVE) regulatory complex (WRC) (7). In immune cells, the WRC consists of multiple subunits including WAVE protein 2 (WAVE2); hematopoietic stem/progenitor cell protein 300 (HSPC300), abelson interacting protein-1 or -2 (Abi1 or -2); hematopoietic protein-1 (Hem-1) (8); and cytoplasmic FMR1 interacting protein-1 (CYFIP1) (9, 10). In response to activation of Rac, the WRC activates the actin regulatory protein 2/3 (Arp2/3) complex, which stimulates actin nucleation and formation of F-actin from monomeric G-actin, leading to the induction of actin regulated processes (11, 12). The importance of properly regulated actin dynamics in immune cells is underscored by observations that variants in the genes that regulate the assembly and disassembly of F-actin, including *WASp* (13)*, WIP* (14), *Rac2* (15), *Moesin* (16), *Dock2* (17), and *Dock8* (18, 19), result in primary immunodeficiency diseases (PID) in humans (see ref. 18 for review). Although PIDs due to variants in WRC components had not been previously realized, 7 independent kindreds were recently identified with PIDs due to loss-of-function variants in Hem-1 (20–22), a hematopoietic cell–specific WRC component (8, 10). Affected children are severely immunodeficient, characterized by dysgammaglobulinemia, poor antipneumococcal and EBV Ab responses following immunization, and increased autoantibodies highly suggestive of dysregulated B cell immunity. However, because of the small number of human patients with PID, extensive genetic heterogeneity, and concurrent diseases and infections (viral meningitis, pneumococcal pneumonia and other recurring respiratory infections, asthma, skin infections, and renal disease), it is extremely difficult to separate secondary effects from primary cell autonomous effects of Hem-1 loss. Similarly, mice with a noncoding point mutation in *Nckap1l* (*Hem1)* are severely immunodeficient, characterized by defective T cell activation, T and B cell lymphopenia, hemolytic anemia, dysregulated cytokine production, defective phagocytosis by macrophages, neutrophil migration defects, failure to thrive, and autoimmunity (23, 24).

In this study, we utilized constitutive and B cell–specific Hem-1–null mice in order to dissect the importance of Hem-1 in B cell development and protective immunity. We demonstrate that B cell disruption of Hem-1 inhibits the development of MZ and B1 cells, resulting in poorly generated TI Ab responses and failed protection against *Streptococcus pneumoniae* (*Spn*). Transitional and mature recirculating B cells fail to migrate efficiently in vitro. Surprisingly, B cell–specific disruption of Hem-1 resulted in increased IgM and IgG2c Ab production in response to immunization with TD Ags, which correlated with hyperresponsive BCR signaling, increased Tbet^+^CD11c^+^ B cells, and increased Ab production. These results suggest that loss-of-function variants of HEM-1 in humans result in poor immune responses against encapsulated bacteria, increased susceptibility to pneumococcal pneumonia, and increased autoantibody production, in part, via B cell–specific mechanisms.

## Results

### Constitutive deletion of Hem-1 severely disrupts B cell development.

**Figure 1:**
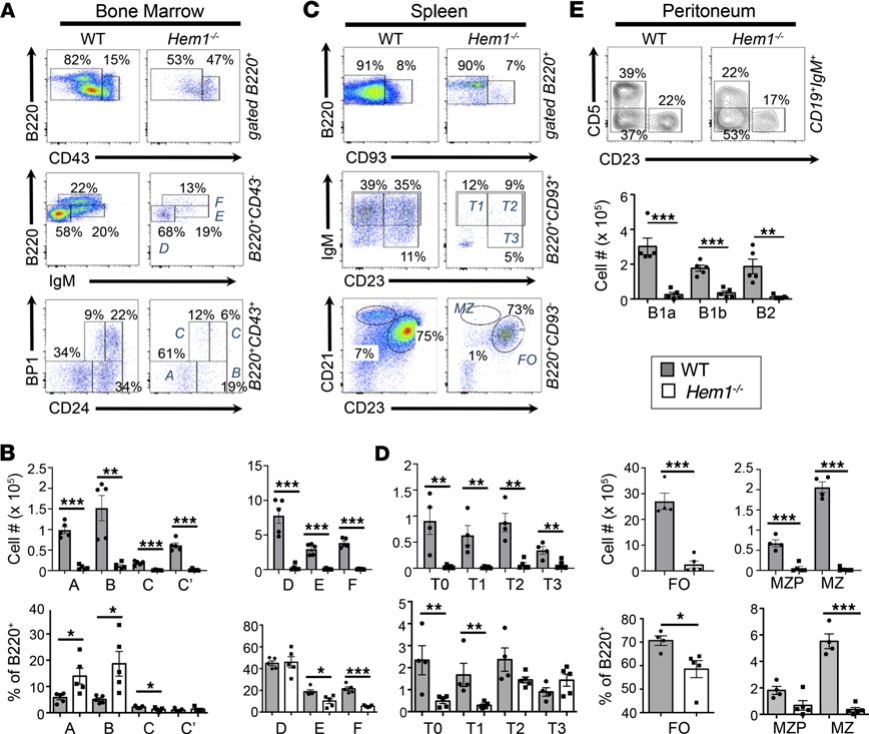
Constitutive disruption of Hem-1 results in impaired B cell development. BM cells, splenocytes, and peritoneal cells were isolated from 6- to 12 week-old *Hem1^–/–^* and littermate control mice. Cells were stained with the indicated fluorescent conjugated antibodies followed by flow cytometric analyses. (**A**) Representative flow cytometric dot plot histograms. (**B**) Bar graphs and quantification of B cell populations (Hardy fractions A–F) in BM cells. (**C**) Representative dot plot histograms. (**D**) Bar graphs with quantification of B cell populations (T0-T3 MZ, FO, MZP, and MZ) isolated from spleens. (**E**) Representative flow cytometric contour histograms and bar graphs showing quantification of B1 and B2 B cells isolated from the peritoneal cavities. Data are representative of ≥ 5 experiments with WT and *Hem1^–/–^* mice (*n* ≥ 10 mice per group). Each data point represents an individual mouse, and the graphs are derived from independent experiments. Data represent mean ± SEM and were analyzed via unpaired Student’s *t* test. *P < 0.05, **P < 0.01, ***P < 0.001.

To investigate how loss-of-function variants of *NCKAP1L* affect the development and functions of specific immune cells*,* we utilized constitutive *Nckap1l* null (*Hem1^–/–^*) and conditional *Nckap1l* floxed (*Hem1^fl/fl^*) alleles in mice using the Cre-*LoxP* system (25). Constitutive disruption of *Nckap1l* in *Hem1^–/–^* mice resulted in profound effects on the development of T lymphocytes and granulocytes, as we previously reported in mice with a noncoding point mutation in *Nckap1l* (data not shown; ref. 23). *Hem1^–/–^* mice were also smaller in size into adulthood (Supplemental Figure 1A; supplemental material available online with this article; https://doi.org/10.1172/jci.insight.153597DS1), consistent with delayed growth and development often noted with patients with severe PID. Examination of BM B cell populations from *Hem1^–/–^* and littermate control mice demonstrated severe B cell lymphopenia starting at the pre-pro–B cell developmental stage (Hardy fraction A) and extending through the mature recirculating FO B cell (B220^hi^CD43^–^IgM^+^; Hardy fraction F) stage (Figure 1, A and B). Peripheral B cell populations in the spleens of *Hem1^–/–^* mice were similarly reduced, including transitional T0 (B220^+^CD93^+^IgM^+^IgD^–^CD23^–^) cells, which are the first emigrants from the BM, T1 (B220^+^CD93^+^IgM^+^IgD^+^CD23^–^), T2 (B220^+^CD93^+^IgM^+^IgD^+^CD23^+^), and T3 (B220^+^CD93^+^IgM^lo^IgD^+^CD23^+^) B cell stages. FO B cells were reduced (B220^+^CD93^–^CD21^+^CD23^+^), with the most pronounced B cell loss occurring in the MZ B cell (B220^+^CD93^–^CD21^hi^CD23^lo^) population (Figure 1, C and D). Long-lived fetal liver–derived peritoneal B1a B cells, BM-derived peritoneal B1b cells, and peritoneal B2 cells were also significantly reduced (Figure 1E). These results recapitulate the B cell phenotype of mice with a noncoding point mutation in *Nckap1l* (Supplemental Figure 1), indicating that constitutive disruption of Hem-1 either by gene deletion or via a single point mutation can profoundly affect B cell development.

### B cell–specific deletion of Hem-1 inhibits the development of transitional, MZ, and mature recirculating FO B cells.

Constitutive tissue-wide loss of Hem-1 in mice and humans results in the dysregulation of multiple hematopoietic cell lineages, which could indirectly disrupt the survival of developing B cells. Hence, to determine the B cell–specific functions of Hem-1, *Hem1^fl/fl^* mice (25) were bred to *Mb1Cre* mice, which express the Cre enzyme under the control of the *Mb1* promoter driving B cell–specific expression beginning at the pro–B cell stage (26). To demonstrate the efficacy of Cre-mediated deletion, *Hem1^fl/fl^Mb1Cre* mice were bred with tdTomato mice, whereby the reporter gene *tdTomato* encoding a red fluorescent protein is expressed following removal of a floxed stop codon by the Cre recombinase (27). This system revealed significant Cre activity (>95%) in B cells throughout B cell development, beginning at the pro–B cell stage in *Hem1^fl/fl^Mb1CretdTomato* mice, indicating that the deletion is likely complete and that Cre-negative or Cre-low expressing cells are not selected for during development (Supplemental Figure 2).

**Figure 2:**
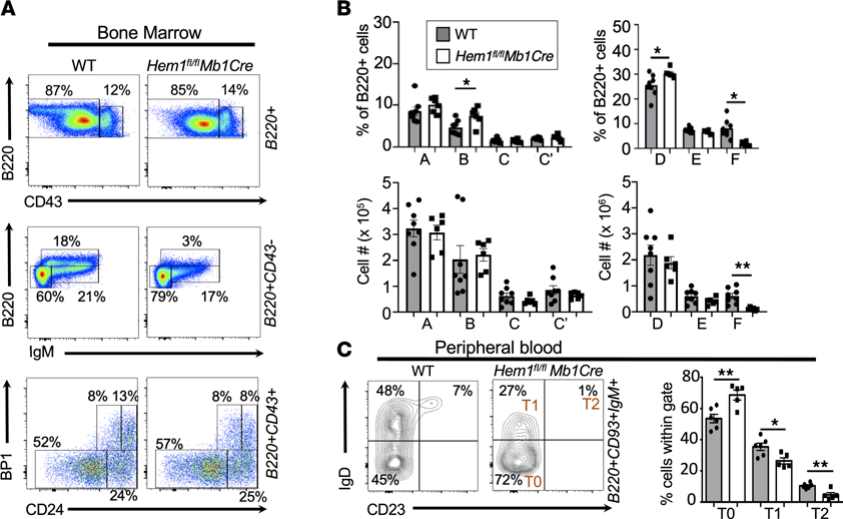
B cell–specific disruption of Hem-1 results in a reduction of mature recirculating follicular B cells. Total BM cells were isolated from BM of 6- to 12-week-old *Hem1^fl/fl^Mb1Cre* and WT littermate mice, followed by staining with fluorescent conjugated antibodies against the indicated surface markers and flow cytometric analyses. (**A**) Representative dot-plot histograms. (**B**) Bar graphs with percentages (top) and cell numbers (bottom) of B cell populations falling within Hardy fractions A–F. Each data point represents individual mice, and graphs are representative of > 5 independent experiments (*n* = 22 and *n* = 20). (**C**) Peripheral blood samples were isolated from WT and *Hem1^fl/fl^Mb1Cre* mice, followed fluorescent Ab staining against the indicated surface markers and flow cytometric analyses. Shown are representative flow cytometric contour histograms (left) and bar graphs with quantification (right) of the proportions of T0 (B220^+^CD93^+^IgM^+^IgD^–^CD23^–^), T1 (B220^+^CD93^+^IgM^+^IgD^+^CD23^–^), and T2 (B220^+^CD93^+^IgM^+^IgD^+^CD23^+^) B cells in peripheral blood. Each data point is representative of a single mouse, and data were collected from 2 independent experiments (*n* = 6 and *n* = 5). Data represent mean ± SEM and were analyzed via unpaired Student’s *t* test. *P < 0.05, **P < 0.01.

Analysis of BM from *Hem1^fl/fl^Mb1Cre* mice revealed that, relative to WT mice, Hem-1–deficient B cells developed relatively normally through the immature B cell (B220^lo^CD43^–^IgM^+^) stage and expressed similar Ig κ/λ light-chain ratios, indicative of normal receptor editing (Figure 2, A and B, and Supplemental Figure 3). In contrast, FO (B220^hi^CD43^–^IgM^+^, Hardy fraction F) B cells were nearly absent (Figure 2, A and B). Analysis of peripheral blood (PB) samples revealed increased representation of transitional T0 (and reduced T1 and T2) B cells in blood from *Hem1^fl/fl^Mb1Cre* mice relative to control mice, consistent with either increased egress of T0 cells from the BM into PB and/or reduced homing of T0 cells to the spleen (Figure 2C). To further address these possibilities, we injected *Hem1^fl/fl^Mb1Cre* and *Hem1^fl/fl^* control mice with anti-CD19 conjugated to phycoerythrin (PE) i.v. and harvested BM, spleen, and PB 2 minutes after injection. It has previously been shown that i.v.-injected anti-CD19PE rapidly equilibrates throughout the BM and spleen and labels B cells present in blood-filled sinusoids but not in parenchymal tissues, where B cells are protected from Ab access shortly after i.v. injection (28). We found an increased representation of *Hem1^fl/fl^Mb1Cre* immature B cells in the BM parenchyma (CD19PE^–^) versus BM sinusoids (CD19PE^+^), consistent with increased retention in the BM parenchyma or increased egress from the BM sinusoids into PB (Supplemental Figure 4). In addition, we found increased representation of *Hem1^fl/fl^Mb1Cre* FO B cells in the BM sinusoid versus BM parenchyma, consistent with reduced abilities of *Hem1^fl/fl^Mb1Cre* FO B cells to enter or be retained in the BM parenchyma relative to WT FO B cells.

**Figure 3:**
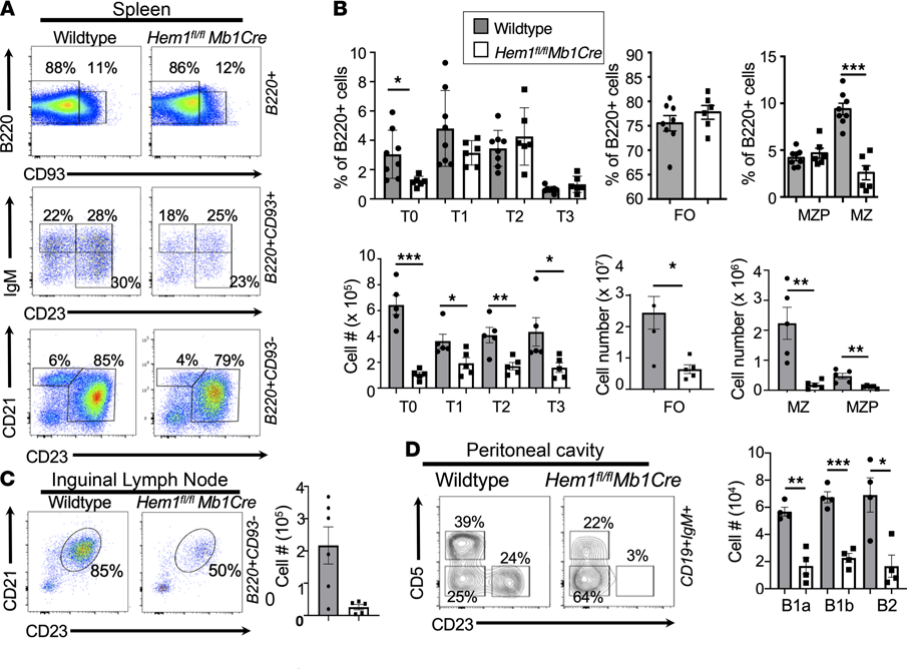
B cell–specific disruption of Hem-1 results in impaired peripheral B cell development. Splenocytes, inguinal LNs, and peritoneal cavity cells were isolated from 6- to 12-week-old WT and *Hem1^fl/fl^Mb1Cre* mice. Cells were stained with fluorescent conjugated antibodies against the surface markers shown, followed by flow cytometric analyses. (**A**) Representative flow cytometric dot plot histograms. (**B**) Bar graphs with quantification of B cell populations isolated from spleens. Each data point represents an individual animal, and the graphs are representative of > 5 independent experiments (*n* = 22 and *n* = 20). (**C**) Representative dot plot histograms (left) and bar graph showing quantification (right) of FO B cells isolated from inguinal LNs (*n* = 6 and *n* = 5). Each data point represents an individual animal, and the graph is representative of > 2 independent experiments. (**D**) Representative contour histograms (left) and bar graph showing quantification of B cell populations isolated from the peritoneal cavity of WT and *Hem1^fl/fl^Mb1Cre* mice (*n* = 4 and *n* = 4). Data represent mean ± SEM and were analyzed via unpaired Student’s *t* test. *P < 0.05, **P < 0.01, ***P < 0.001.

We next assessed whether B cell–specific disruption of Hem-1 affected the representation and maturation of B cells in the peripheral lymphoid tissues. Analyses of the spleens from *Hem1^fl/fl^Mb1Cre* mice revealed that the percentage of transitional T0 cells was decreased, as was the total number of transitional T0, T1, T2, and T3 B cells in *Hem1^fl/fl^Mb1Cre* mice relative to control mice (Figure 3, A and B). Similar to what was observed in *Hem1^–/–^* mice, FO B cells were reduced and MZ B cells were almost completely absent. FO B cells in inguinal lymph nodes (LNs) of *Hem1^fl/fl^Mb1Cre* mice were also reduced (Figure 3C), as were long-lived resident B1a and B1b B cells in the peritoneum (Figure 3D) and spleen (Supplemental Figure 3). These results demonstrate the B cell autonomous importance of Hem-1 in the development and/or homeostasis of both conventional and innate-like B cells.

### Disruption of Hem-1 inhibits B cell migration.

**Figure 4:**
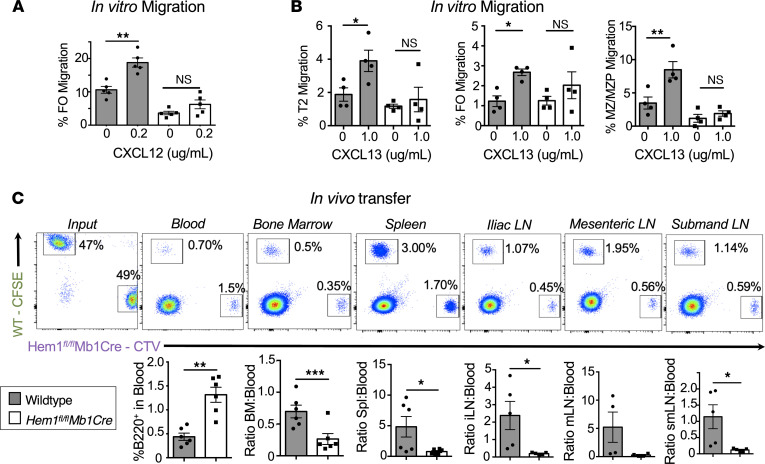
Conditional deletion of Hem-1 in B cells disrupts B cell migration and homing to lymphoid tissues. In vitro migration of splenic B cells isolated from WT and *Hem1^fl/fl^Mb1Cre* mice in transwell plates following stimulation with 0.2 μg/mL CXCL12 or 1 μg/mL CXCL13. (**A**) Shown are bar graphs depicting the percent FO B cells migrated. Data points are representative of individual mice from a single experiment. Data are representative of 2 individual experiments (*n* = 8 and *n* = 8). (**B**) Shown are bar graphs depicting the percent T2, FO, and MZ/MZP cells migrated in the absence or presence of CXCL13 stimulation. Data points are representative of individual mice from a single experiment. Data are representative of 2 individual experiments (*n* = 7 and *n* = 7). (**C**) WT CFSE-labeled B cells were mixed 1:1 with CTV-labeled *Hem1^fl/fl^Mb1Cre* B cells combined at a 1:1 ratio and injected into WT host mice. The representation of WT and *Hem1^fl/fl^Mb1Cre* B cells in the input (far left) and recipient tissues (right) were determined by flow cytometry 24 hours after i.v. injection. Shown are representative dot plot histograms (top) and graphical representations (bottom) of the ratios of B220^+^ B cells in peripheral blood versus each respective tissue in individual recipient mice. The data are representative of 2 independent experiments of recipient mice (*n* = 10). Data represent the mean ± SEM and were analyzed via an paired Student’s *t* test (**A** and **B**) and unpaired Students *t* test (**C**). *P < 0.05, **P < 0.01, ***P < 0.001.

Actin polymerization has been shown to be important for the generation of filopodia and lamellipodia, which drive cell migration (29). We hypothesized that disruption of Hem-1 resulted in impaired migration into essential lymphoid niches. Using transwell migration plates, we found that Hem-1–deficient B cells, and FO B cells in particular, are deficient in their abilities to migrate in response to the chemokine CXCL12, which is recognized by the chemokine receptor 4 (CXCR4) on the surface of immune cells, including B cells (Figure 4A). CXCR4 was equally expressed on B cells from *Hem1^fl/fl^Mb1Cre* and WT mice, indicating that the reduced ability to migrate efficiently was not due to reduced chemokine receptor expression on B cells (Supplemental Figure 3B). Hem-1–deficient T2, FO, and MZ/marginal zone precursor (MZ/MZP) B cells also responded poorly to CXCL13 (a chemokine that regulates B cell homing to lymphoid follicles), whereas control B cell migration was significantly increased (Figure 4B).

We next explored the abilities of Hem-1–deficient B cells to home from PB into lymphoid tissues, such as BM, spleen, and LNs. We utilized a competitive in vivo transfer approach whereby purified *Hem1^fl/fl^Mb1Cre* and WT B cells were labeled with the fluorescent dyes Cell Trace Violet (CTV) and CFSE, respectively, mixed 1:1, and transferred via i.v. injection into recipient control mice. Twenty-four hours after transfer, recipient mice were euthanized, and the ratios of labeled WT versus Hem-1–deficient B cells in recipient mice were determined in PB and lymphoid tissues. We found that *Hem1^fl/fl^Mb1Cre* B cells made up a larger proportion of B cells in PB of recipient mice 24 hours after injection, despite being injected at a 1:1 ratio (Figure 4C). In addition, the proportion of *Hem1^fl/fl^Mb1Cre* B cells versus WT B cells found in peripheral lymphoid tissues, including BM, spleen, iliac LN, mesenteric LN (MLN), and submandibular LN, were decreased relative to PB, consistent with decreased B cell migration and/or retention from PB into lymphoid tissues following disruption of Hem-1.

### Disruption of Hem-1 inhibits TI Ab production and increases susceptibility to Spn challenge.

**Figure 5:**
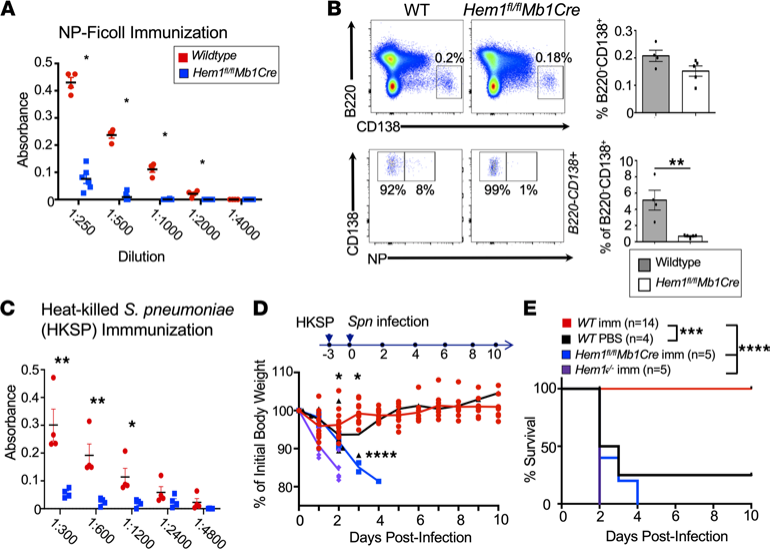
Hem-1 is required for optimal TI Ab production and protection against *Streptococcus pneumoniae* challenge. (**A** and **B**) WT and *Hem1^fl/fl^Mb1Cre* mice (*n* = 4 and *n* = 5) were immunized with 50 μg/mL NP-Ficoll. (**A**) ELISA plates were coated with NP-BSA_30_ (10 μg/mL), and Ab production was measured 6 days after immunization. Shown is a graph depicting absorbance at 405 nm versus serum dilution. (**B**) BM was harvested from immunized mice 6 days after immunization. Cells were stained for fluorescent conjugated antibodies against the indicated markers for flow cytometric analyses. Shown are representative dot plot histograms and bar graphs quantifying the percentages of total plasma cells (B220^–^CD138^+^) (top) and NP-specific plasma cells (bottom). Each data point is representative of an individual mouse, and data were derived from a single experiment. (**C**) ELISA plates were coated with 50 μg/mL PC-BSA and used to determined IgM production 5 days after i.v. immunization with 1 × 10^7^ cfu heat-killed *Streptococcus pneumoniae* (HKSP). Shown are graphs depicting the absorbance at 405 nm versus serum dilution. (**D** and **E**) WT, *Hem1^–/–^*, and *Hem1^fl/fl^Mb1Cre* mice (*n* = 14, 5, and 5) were immunized with HKSP. Immunized (imm) mice, and unimmunized WT control mice (*n* = 4), were challenged with 1 × 10^7^ cfu live *Spn* and were monitored daily. (**D**) A graph depicting BW loss versus days after infection. (**E**) A Kaplan-Meier survival curve for each group. Data points represent individual mice, and data were combined from 2 independent experiments. Data represent mean ± SEM and were analyzed via unpaired Student’s *t* test (**A**–**C**) or the Gehan-Breslow-Wilcoxon test using GraphPad prism (**E**). Shown are individual comparisons. For multiple comparisons using ANOVA, *P* < 0.0001. *P < 0.05, **P < 0.01, ***P < 0.001, ****P < 0.0001.

Innate-like MZ and B1 B cells are important for early Ab responses to TI antigens (e.g., those found in encapsulated bacteria such as *Spn*, an important community-acquired pathogen). Because *Hem1^fl/fl^Mb1Cre* mice have decreased MZ and B1 B cells, we hypothesized that *Hem1^fl/fl^Mb1Cre* mice would have impaired TI Ab responses following immunization. We immunized *Hem1^fl/fl^Mb1Cre* and WT mice with the TI type II antigen 4-hydroxy-3-nitrophenyl-acetyl (NP) conjugated to Ficoll. Six days following immunization, sera and tissues were collected, and IgM titers against a portion of the NP immunogen (NP30-BSA) were measured by ELISA. We found that *Hem1^fl/fl^Mb1Cre* mice produced little or no IgM against the NP, whereas WT mice mounted normal responses (Figure 5A). Although total B220^lo^CD138^+^ plasma cells were only marginally reduced in *Hem1^fl/fl^Mb1Cre* mice, NP-specific plasma cells were reduced ~6-fold relative to WT mice (Figure 5B).

Children with mutations in *NCKAP1L* generate poor TI Ab responses following immunization against *Spn* (20). Thus, we next assessed the Ab responses of Hem-1–deficient and WT mice to heat-killed *Spn* (HKSP). We found that *Hem1^fl/fl^Mb1Cre* mice failed to produce significant amounts of IgM Abs against phosphocholine, a component of the *Spn* cell surface, conjugated to BSA (PC-BSA) 3 days following immunization with HKSP (Figure 5C). To determine whether the poor TI response altered protection against *Spn* infection, we immunized WT, *Hem1^fl/fl^Mb1Cre*, and *Hem1^–/–^* mice with HKSP. The mice were then challenged with a lethal dose of *Spn* (serotype 2 strain D39) 3 days after immunization. We found that all infected mice showed signs of infection and lost BW on day 1 after infection (Figure 5D). On day 2 after infection, all immunized *Hem1^–/–^* mice, 3 of 5 immunized *Hem1^fl/fl^Mb1Cre* mice, and 2 of 4 unimmunized WT mice either died or reached endpoint criteria and were euthanized (Figure 5E). The remaining immunized *Hem1^fl/fl^Mb1Cre* mice continued to lose BW and reached endpoint criteria by day 4 after infection, whereas all immunized WT mice regained normal BW and survived (Figure 5, D and E). These results indicate that B cell–specific expression of Hem-1 is essential for generating protective TI Ab responses against pathogenic *Spn*.

### Hem-1 deficiency in B cells results in increased IgM and IgG2c Ab and GC responses following immunization with TD antigens.

**Figure 6:**
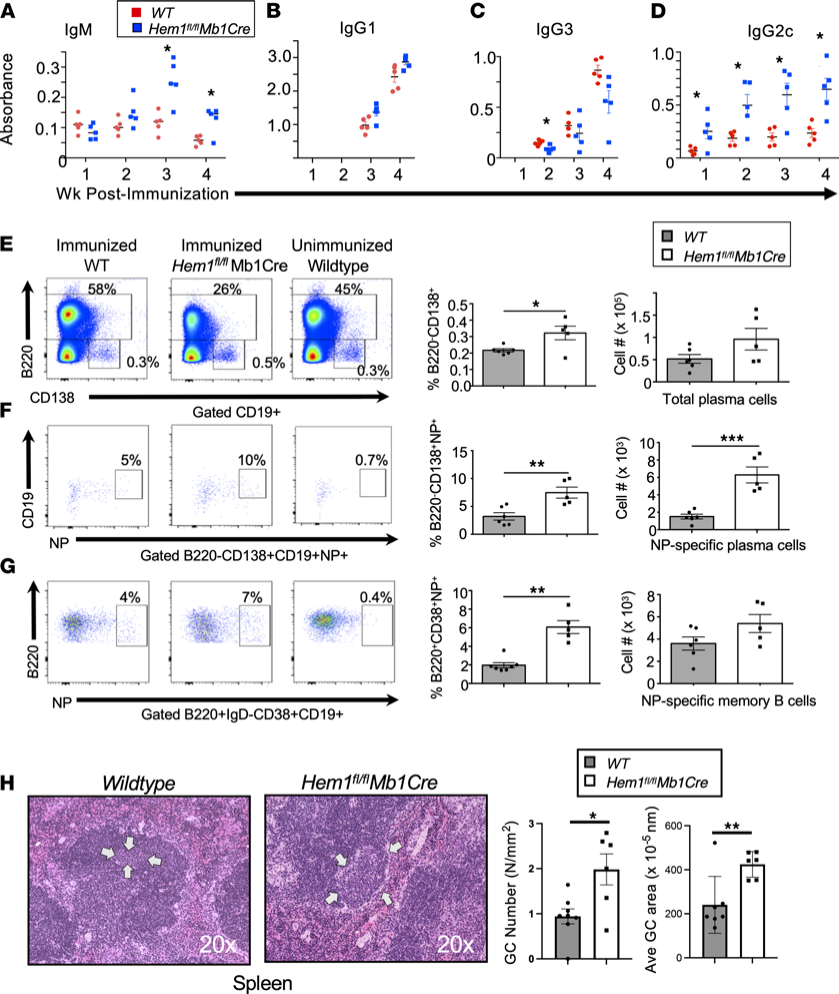
Conditional deletion of Hem-1 in B cells results in increased IgM and IgG2c Ab production in response to immunization with TD Ags. Eight- to 10-week-old mice were immunized with 100 μg/mouse NP-KLH in alum via i.p. injection. (**A**–**D**) Shown are graphs depicting NP-specific IgM, IgG1, IgG3, and IgG2c production based on OD at 405 nm over a 4-week period after immunization. *n* = 5 mice per group. Each data point represents a single mouse and is representative of a single experiment. Data represent mean ± SEM and were analyzed via unpaired Student’s *t* test. *P < 0.05. (**E**–**G**) WT and *Hem1^fl/fl^Mb1Cre* mice were immunized with NP-KLH. Four weeks after immunization, mice were boosted with 20 μg/mouse NP-KLH in PBS. Seven days after boost, splenocytes were harvested and cells were stained for flow cytometric analyses. (**E**) Shown are representative dot plot histograms (left) and graphs with quantification of total plasma cells (B220^–^CD138^+^) (right) isolated from immunized WT and *Hem1^fl/fl^Mb1Cre* mice and unimmunized control mice. (**F**) Shown are representative dot plot histograms (left) and quantification of NP-specific plasma cells (B220^–^CD138^+^CD19^–^NP^+^) (right) from immunized WT and *Hem1^fl/fl^Mb1Cre* mice and unimmunized control mice. (**G**) Representative flow cytometric histograms and graphs with quantification of memory B cells (B220^+^IgD^–^CD38^+^CD19^+^NP^+^) from immunized WT, and *Hem1^fl/fl^Mb1Cre* mice and unimmunized control mice. (**H**) H&E-stained spleen sections from individual mice showing germinal center size (20× magnification) (left) and bar graphs depicting GC number/area and average GC area (right). Arrows denote the size of representative GCs. Each data point is representative of an individual mouse and is representative of a single experiment. Data represent mean ± SEM and were analyzed via unpaired Student’s *t* test. *P < 0.05, **P < 0.01, ***P < 0.001.

Unlike the B cells in the peritoneum or MZ, FO B cells require interactions with Tfh cells to produce Abs against TD antigens such as NP– keyhole limpet hemocyanin (NP-KLH). To test the abilities of Hem-1–deficient B cells to respond to TD antigens, *Hem1^fl/fl^Mb1Cre* and WT mice were immunized with NP-KLH in alum, and Ab responses against NP30-BSA were measured on a weekly basis for 4 weeks. In contrast to Ab responses to TI antigens, *Hem1^fl/fl^Mb1Cre* mice initially produced normal levels of NP-specific IgM Abs 1 and 2 weeks after immunization, and NP-specific IgM levels increased on weeks 3 and 4 after immunization to levels above that produced by WT mice (Figure 6A). *Hem1^fl/fl^Mb1Cre* mice also produced similar quantities of IgG1 and slightly reduced levels of IgG3 (Figure 6, B and C). Interestingly, IgG2c production was significantly increased in *Hem1^fl/fl^Mb1Cre* relative to WT mice as early as 1 week after immunization and remained higher than control mice up through 4 weeks after immunization (Figure 6D).

To determine the importance of Hem-1 in the generation of antigen-specific plasma cells and memory B cells, WT and *Hem1^fl/fl^Mb1Cre* mice were immunized i.p. with NP-KLH in alum. Four weeks after immunization, mice were boosted with NP-KLH in PBS. Eight days following boost immunization, the spleens were collected and the representation of memory B and plasma cells were assessed (30). Relative to WT mice, *Hem1^fl/fl^Mb1Cre* mice had increased percentages of total plasma cells (Figure 6E) and increased percentages and total numbers of NP-specific plasma cells (B220^–^CD138^+^CD19^–^NP^+^) (Figure 6F). NP-specific memory B cells (B220^+^IgD^–^CD38^+^CD19^+^NP^+^) generated in response to boost immunization were also elevated in *Hem1^fl/fl^Mb1Cre* relative to WT control mice (Figure 6G). This correlated with increased splenic GC number and size, as determined by histological analyses (Figure 6H).

To test the abilities of *Hem1^fl/fl^Mb1Cre* mice to generate GCs and plasma cells in response to a model respiratory virus, WT and *Hem1^fl/fl^Mb1Cre* mice were infected with the mouse adapted PR/8 influenza A virus (IAV). Ten days after infection, mediastinal LNs were collected and analyzed for GC (B220^+^CD95^+^GL7^+^), plasmablast (B220^+^IgD^–^CD138^int^) and plasma cell (B220^+^IgD^–^CD138^hi^) formation (31). Both WT and *Hem1^fl/fl^Mb1Cre* mice lost equivalent body weight and did not demonstrate obvious differences in susceptibility to the IAV (data not shown). However, a greater percentage of *Hem1^fl/fl^Mb1Cre* B cells formed GCs and differentiated into plasmablasts and plasma cells within mediastinal LNs draining the lungs relative to B cells from IAV-infected WT control mice (Supplemental Figure 5A). To further probe antigen-specific GC responses, we immunized WT and *Hem1^fl/fl^Mb1Cre* mice with virus-like particles (VLPs), which are a specific class of subunit immunogens that mimic the structures of authentic virus particles. Fourteen days after immunization, splenocytes and MLN were harvested, and total GCs, VLP specific GCs, and Tfh cells were assessed by flow cytometry. Whereas total splenic B cells were reduced in *Hem1^fl/fl^Mb1Cre* relative to littermate control mice, the representation of VLP-specific B cells, total GCs, and VLP-specific GCs were all increased (Supplemental Figure 6A). In contrast, the representation and total numbers of CD4^+^CXCR5^+^PD1^+^intracellular BCL6^+^ Tfh cells relative to CD4 T cells were equivalent between *Hem1^fl/fl^Mb1Cre* relative to littermate control mice in response to immunization with VLP or 8 days following immunization with sheep RBCs (sRBCs) (Supplemental Figure 6, B and C). These results suggest that disruption of Hem-1 results in increased potential for B cells to generate PB, PC, and GC B cells in response to multiple TD antigens.

### Hem-1 deficiency results in increased autoantibody levels and a higher proportion of Tbet^+^CD11c^+^ ABC cells.

**Figure 7:**
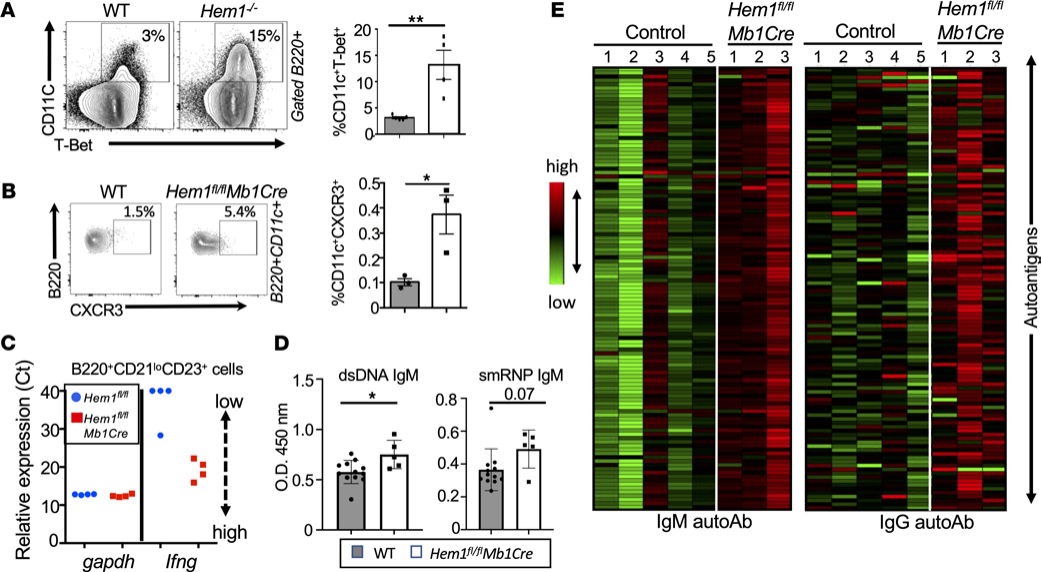
B cell–specific disruption of Hem-1 results in increased autoantibody production and a higher proportion of CD11c^+^T-bet^+^ ABC cells. (**A** and **B**) Representative flow cytometric contour histograms and graphical representation of B220^+^CD11c^+^T-bet^+^ splenic B cells from WT and *Hem1^–/–^* mice, and B220^+^CD11c^+^CXCR3^+^ B cells from WT and *Hem1^fl/fl^Mb1Cre* mice. (**C**) Real-time PCR dot plots showing *gapdh* (loading control) and *Ifng* gene expression (Ct) from B220^+^CD21^lo^CD23^+^ B cells sorted by FACS from WT (blue dots) and *Hem1^fl/fl^Mb1Cre* (red squares) mice and then stimulated for 12 hours with 10 μg/mL anti-IgM and 20 U/mL IFN-γ**.** Relative gene expression increases as Ct decreases. (**D**) Sera were collected from M and F mice ages 13–28 weeks. Shown are graphs depicting isotype-specific anti-dsDNA and anti-smRNP autoantibodies as determined by ELISA. Each data point represents an individual mouse, and the data were collected from a single experiment. (**E**) Sera were collected from female *Hem1^fl/fl^Mb1Cre* and control mice ages 28–32 weeks. Sera were then hybridized to an autoantigen microarray containing 128 antigens. Shown are heatmaps depicting antigen reactivity (antibody score) for IgM (left) and IgG (right). Statistically significant changes in female mice are noted in Supplemental Figure 7. Data represent mean ± SEM and were analyzed via unpaired Student’s *t* test or paired Student’s *t* test. *P ≤ 0.05, **P ≤ 0.01, ***P ≤ 0.001.

Increased IgG2c (B6 mice), IgG2a (Balb/c mice), or IgG1/3 (in humans) have been associated with autoimmunity in both mice and humans (32, 33). Class switching to IgG2a/c (IgG1/3 in humans), memory B cell survival, and PC differentiation (34) is induced by the transcription factor T-bet (35–40). A unique population of memory B cells expressing T-bet and CD11c (denoted age-associated B cells [ABC cells]) are known to expand following chronic microbe stimulation and are elevated in many autoimmune diseases, including systemic lupus erythematosus (SLE) (41–45). T-bet expression also controls gene expression of IFN-γ and homing receptor CXCR3 (46, 47). Flow cytometric analyses of *Hem1^–/–^* splenic B cells revealed that constitutive disruption of Hem-1 resulted in increased representation of B220^+^CD11c^+^T-bet^+^ B cells (Figure 7A). Analyses of *Hem1^fl/fl^Mb1Cre^+^* and WT mice indicated that B cell–specific disruption of Hem-1 also resulted in increased expression of CD19^+^T-bet^+^ splenic B cells (Supplemental Figure 5B), as well as increased representation of B220^+^CD11c^+^ B cells expressing CXCR3, a transcriptional target of T-bet (Figure 7B). Quantitative PCR (qPCR) analyses of FACS purified B220^+^CD23^+^CD21^lo^ B cells activated with anti-IgM and IFN-γ for 12 hours revealed greatly increased *Ifng* expression by *Hem1^fl/fl^Mb1Cre* B cells relative to littermate *Hem1^fl/fl^* control B cells, which either did not express *Ifng* (3 of 4 mice) or expressed low levels (1 of 4 mice) (Figure 7C). These results suggest that disruption of Hem-1 results in increased representation of ABC-like T-bet^+^ B cells and B cell–derived *Ifng*, which have previously been linked to autoantibody production and autoimmunity.

Hem-1–deficient children were found to produce increased autoantibodies against dsDNA (21, 22) and nuclei (22), and several patients were diagnosed with SLE (21) or SLE-like disease (20). Using ELISA assays on serum from *Hem1^fl/fl^Mb1Cre* and WT mice, we found that *Hem1^fl/fl^Mb1Cre* mice produced higher levels of anti-dsDNA relative to WT mice, and anti-smRNP autoantibodies appeared increased but did not quite reach statistical significance (Figure 7D). To examine the spectrum of autoantibody differences in more detail, we profiled autoantibodies against 128 autoantigens in serum from female *Hem1^fl/fl^Mb1Cre* and age-matched WT mice (ages 38–42 weeks), using autoantigen microarray technology. We found that 33 IgM autoantibodies and 38 IgG autoantibodies were significantly increased in sera from *Hem1^fl/fl^Mb1Cre* versus control mice (Figure 7E and Supplemental Figure 7).

Signaling through the Baff (a B cell activating factor belonging to the TNF family) is one of the main prosurvival signals in B cells. B cell lymphopenia can promote increased Baff release, and excess Baff can promote the development of autoreactive B cells (48). Because Hem-1–deficient mice are B cell lymphopenic, we assessed serum Baff levels in *Hem1^fl/fl^Mb1Cre* versus littermate control mice. As expected, disruption of Hem-1 resulted in a ~40% increase in Baff levels (Supplemental Figure 5C), consistent with a feedback response to B lymphopenia. These results collectively suggest that B cell–specific expression of Hem-1 is important for limiting IgM and IgG autoantibody production to a large variety of autoantigens, perhaps in part by limiting the production of ABC cells and excess Baff signaling.

### Hem-1 deficiency results in B cell hyperactivation.

**Figure 8:**
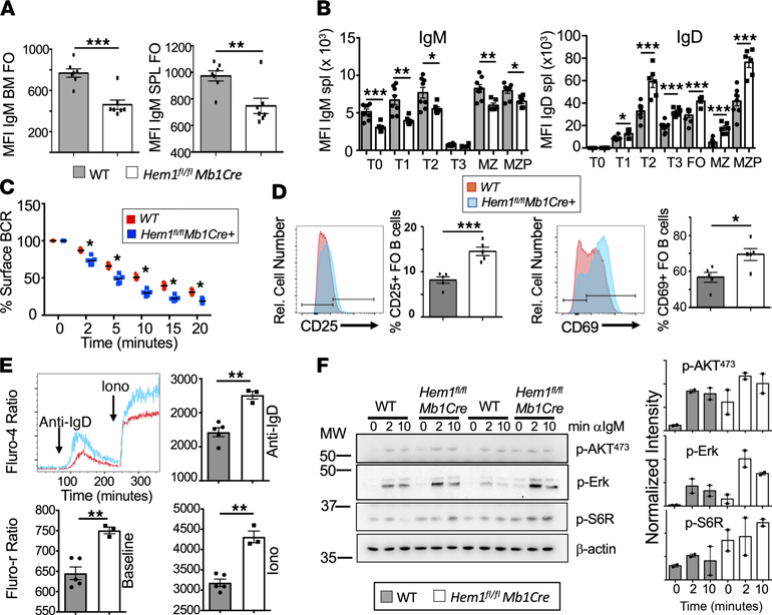
Conditional deletion of Hem-1 in B cells results in hyperresponsive B cell signaling. (**A** and **B**) Total BM cells and splenocytes were isolated from WT and *Hem1^fl/fl^ Mb1Cre* mice and stained with fluorescent Abs followed by flow cytometric analyses. Shown are bar graphs depicting IgM MFI on BM FO (B220^hi^CD43^–^IgM^+^) (left) and mature splenic FO B cells (B220^+^CD93^–^CD21^lo^CD23^hi^) (right) (*n* = 7/group), and MFI of IgM (left) and IgD (right) on splenic B cells during each stage of B cell development (*n* = 8, *n* = 6). (**C**) B220^+^ cells were treated with 10 μg/mL anti-IgM. The MFI at each time point was determined via flow cytometry and compared with the MFI at 0 minutes to determine percent IgM remaining on the surface. *n* = 4 mice/group and represents a single experiment. (**D**) Total splenocytes were treated with 10 μg/mL anti-IgM for 16 hours. Cells were stained with fluorescent antibodies, followed by flow cytometric analyses. Shown are representative histograms and bar graphs showing expression of CD25 (left) and CD69 (right) on gated FO B cells following stimulation. The data were collected from a single experiment (*n* = 5/group). (**E**) Splenic B cells were stained with Fluo-4 and stimulated with anti-IgD, followed by ionomycin to determine BCR induced calcium influx. Data are representative of a single experiment (*n* = 5, *n* = 3). (**F**) Purified B cells were stimulated for 0, 5, and 10 minutes with anti-IgM and activation of signaling molecules were determined by immunoblotting. See complete unedited blots in the supplemental material. Relative intensity was determined using ImageJ and are representative of 2 independent experiments from pooled B cells (*n* = 2 mice/ group). For **A**–**E**, data points are representative of single mice. Data represent mean ± SEM and were analyzed via unpaired Student’s *t* test. *P < 0.05, **P < 0.01, ***P < 0.001.

The generation of T-bet^+^ ABC cells is promoted by synergistic BCR/TLR, IL-21, and IFN-γ signaling and arises following antigen-driven B cell activation (37, 49–52). We hypothesized that, in the absence of Hem-1 and efficient F-actin polymerization, BCR expression may be elevated, resulting in increased BCR signaling, as has been shown for WASp-deficient B cells (53). Surprisingly, B cells from *Hem1^fl/fl^Mb1Cre* mice expressed lower levels of IgM on the cell surface throughout B cell development (Figure 8, A and B, and Supplemental Figure 9A), and IgM expression was further downregulated (internalized) at a greater rate relative to WT B cells following anti-IgM stimulation (Figure 8C).

It has previously been shown that IgM, but not IgD, is downregulated in autoreactive B cells. Relative to IgM, IgD antigen engagement has been shown to result in lower signal strength, and increased expression of IgD at the expense of IgM has been proposed as a mechanism for B cells to escape BCR-mediated deletion and to become more tolerant to self-antigens (54, 55). We hypothesized that decreased IgM expression in Hem-1–deficient B cells could be a mechanism for avoidance of deletion due to excessive signaling (54). Consistent with this hypothesis, we found that expression of IgD was increased in transitional, FO, MZP, and MZ B cells from *Hem1^fl/fl^Mb1Cre* mice relative to *Hem1^fl/fl^* control mice (Figure 8B and Supplemental Figure 9A).

To determine if BCR signaling strength was altered in Hem-1–deficient B cells, we assessed surface levels of the early activation markers CD69 and CD25 before and after anti-IgM or anti-IgD stimulation using flow cytometry. We found that B220^+^ B cells from experimentally naive *Hem1^fl/fl^Mb1Cre* mice expressed significantly higher levels of CD69 and CD25 both basally and 4 hours following anti-IgM or anti-IgD stimulation relative to *Hem1^fl/fl^* littermate control mice (Supplemental Figure 8). CD25 levels were also increased in Hem-1–deficient B cells 24 hours following anti-IgM or anti-IgD stimulation relative to WT B cells (Supplemental Figure 8). Analyses of Hem-1–deficient FO B cells revealed that both CD25 and CD69 levels were increased after 16 hours of anti-IgM stimulation relative to WT FO B cells (Figure 8D). No differences in CD69 and CD25 expression were noted between *Mb1Cre* and WT B cells at 0, 4, and 24 hours following anti-IgM stimulation, indicating that *Mb1Cre* alone is not impacting B cell activation (data not shown). These results collectively suggest that B cell–specific disruption of Hem-1 results in increased B cell activation both basally and following acute IgM or IgD stimulation.

To further examine the consequences of Hem-1 deficiency on intracellular signaling pathways downstream of the BCR, we assessed levels of baseline intracellular calcium and intracellular calcium influx following anti-IgM, -IgD, or ionomycin control stimulation of B cells from *Hem1^fl/fl^Mb1Cre* mice relative to WT mice. Baseline intracellular calcium levels, as well as calcium influx following anti-IgD and ionomycin, were significantly increased in B cells from *Hem1^fl/fl^Mb1Cre* mice relative to littermate control mice (Figure 8E and Supplemental Figure 9B), suggesting that membrane proximal signaling is altered. Calcium influx following anti-IgM stimulation appeared increased, although the differences were not statistically significant (Supplemental Figure 8C). Immunoblot analyses revealed that *Hem1^fl/fl^Mb1Cre* B cells exhibited increased mTORC2 (p-AKT^473^), mTORC1 (p-S6R), and ERK (p-ERK) signaling both basally and following anti-IgM stimulation (Figure 8F), despite lower surface IgM expression (Figure 8B). These results collectively suggest that B cell–specific disruption of Hem-1 results in increased basal B cell activation and hyperresponsiveness to BCR stimulation (56).

## Discussion

Children with PID due to loss-of-function mutations in the *NCKAP1L* gene encoding for Hem-1 suffer from a variety of clinical manifestations, including recurring bacterial and viral infections, pneumonia, poor specific Ab responses, and autoimmunity resulting in high mortality. Similarly, mice lacking Hem-1 in all tissues either due to gene targeting or a noncoding point mutation similarly demonstrated severe immunodeficiency disease, hyperinflammation, autoimmunity, and high mortality rates. In this study, we utilized the Cre-LoxP system to investigate the B cell–specific roles of Hem-1 in protective immunity and autoimmunity. Our results demonstrate that B cell–specific expression of Hem-1 is important for the optimal development of innate-like B1 and MZ B cells, B cell migration in response to CXCL12 and CXCL13, generation of TI Ab responses, and Ab-mediated protection against fatal *Spn* challenge. In addition, our studies reveal that B cell–specific expression of Hem-1 helps limit B cell hyperactivation, class switching to IgG2c, and autoreactivity to self-antigens. These results collectively suggest that the severe immunodeficiency disease and autoimmunity characteristic of Hem-1–deficient patients with PID may be due, in part, to the loss of Hem-1 and the WRC specifically in B cells.

Hem-1–deficient children respond poorly to immunization against *Spn* and are highly susceptible to Pneumococcal pneumonia, a common community-acquired disease. Here, we found that B cell–specific disruption of Hem-1 in mice resulted in a reduction of innate-like MZ and B1 B cell populations and failed Ab production in response to immunization with the TI antigens NP-Ficoll and HKSP. Immunization of B cell–specific Hem-1–deficient mice with HKSP failed to protect against lethal *Spn* challenge, whereas WT controls were completely protected. These results suggest that a deficiency of Hem-1 and the WRC in B cells may be partially responsible for why Hem-1–deficient children respond poorly to Pneumovax immunization and exhibit increased susceptibility to encapsulated pathogens such as *Spn*.

We also found that B cell–specific disruption of Hem-1 resulted in a reduction of mature recirculating FO B cells in the BM and LNs. In addition, we found a reduction of all B cell subsets in the spleen, beginning at the transitional T0 stage. During B cell development, immature transitional T0 cells migrate from the BM to the spleen, where B cell entry into the red pulp and white pulp are critical steps in normal B cell maturation and central tolerance (57). In particular, the activity of Rac GTPases, which activate the WRC in response to immune receptor activation, has previously been shown to be important for the migration of T0 B cells from PB to the splenic white pulp, where B cell development continues (58). In our study, we found a higher proportion of T0 B cells in PB of *Hem1^fl/fl^Mb1Cre* mice relative to WT mice, and this correlated with a reduction in T0 cells in spleen. These results suggest that Hem-1–deficient T0 cells may have failed to effectively migrate and/or be retained in peripheral lymphoid niches, resulting in reduced B cell maturation. Importantly, Hem-1–deficient patients with PID were also found to have reduced transitional B cell populations (21), perhaps reflecting dysregulated B cell development and migration in humans, as well.

Using transwell migration plates, we found that FO B cells from *Hem1^fl/fl^Mb1Cre* failed to migrate in response to CXCL12, and T2, FO, and MZ/MZP B cells failed to migrate in response to CXCL13, whereas control B cells migrated normally. CXCL13, in particular, is important for directing migration of transitional B lymphocytes into the white pulp, and FO B cells to the BM (58). Using a mixed chimera approach in vivo, we found that Hem-1–deficient B cells failed to compete with WT B cells for homing or retention from PB into lymphoid tissues including spleen, BM, and MLNs, iliac LNs, and submandibular LNs. Using a CD19PE injection strategy, we found that FO B cells from *Hem1^fl/fl^Mb1Cre* mice failed to efficiently enter and/or be retained in the BM parenchyma and remained in the BM sinusoids. These results support our hypothesis that the reduction of FO B cells in the BM and LN may be due, in part, to impaired B cell homing and/or retention into lymphoid tissues. Consistent with this notion, Hem-1–deficient primary murine macrophages migrated poorly and accumulated in blood vessels in vivo due to defective lamellipodial protrusions (59); Hem-1–deficient primary T cells (20), and murine and human neutrophils, migrated poorly both in vitro and/or in vivo (20, 25).

One of the most consistent phenotypes of Hem-1–deficient children is the presence of dsDNA autoantibodies and autoimmune disease, including immune complex glomerulonephritis, SLE, and SLE-like disease (20, 21). In addition, Hem-1–deficient children often present with signs of hyperinflammation, including increased ferritin and soluble IL-2 receptor, liver calcification (20), splenomegaly, gastroenteritis, and hemophagocytic lymphohistiocytosis (20, 22), which collectively contribute to growth abnormalities and failure to thrive. In our study, we also found increased anti-dsDNA Abs via ELISA in *Hem1^fl//fl^Mb1Cre* versus WT mice. In addition, using autoantigen microarrays, we found that B cell–specific disruption of Hem-1 resulted in increased IgM and IgG autoantibodies targeting a large spectrum of autoantigens, including core nucleosome components (histones H2A, H3, and H4; core histone; and nucleosomal antigen) and ribonucleoproteins (U1-snRNAP 68/70, U1-snRNP B/B’, smRNP, SmD1, SmD3, Sm), which are associated with SLE in humans (60). Similar to Hem-1–deficient children, Hem-1–null mice exhibited membranous glomerulopathy (23), a disease often associated with autoantibody production; increased expression of the acute phase proteins serum amyloid A1 (SAA1), SAA2, and SAA3; splenomegaly; anemia; liver calcification (due to amyloid deposition); and diffuse inflammation in multiple tissues including epididymis, mesentery, heart, and lungs (23, 24). Hem-1–deficient mice were also consistently smaller in size, suggesting that Hem-1 loss in hematopoietic cells is sufficient to disrupt overall organismal growth in both mice and humans. These results collectively suggest that Hem-1 is essential for limiting hyperinflammation and enforcing B cell tolerance.

It had previously been shown that the IgG2a/c (IgG1/3 in humans) subclass is overrepresented in autoantibodies, and that class switching to IgG2a/c, memory B cell survival, GC formation, tissue localization (61–63), and PC differentiation (34) are regulated by the transcription factor T-bet (35–39). CD21^–/lo^CD11c^+^CXCR3^+^ ABC cells expressing T-bet are increased in SLE (64, 65), Crohn’s disease (66), Sjogren’s syndrome (67), rheumatoid arthritis (52), common variable immunodeficiency (68, 69), and multiple sclerosis (70). In a cohort of over 200 SLE patients, the degree of T-bet^+^CD11c^+^ B cell expansion positively correlated with the severity of disease (65). In the murine SLE models, CD11c^+^T-bet^+^ ABC cells are required for antichromatin Ab production (52, 71), and targeted disruption of *the Tbx21* gene encoding T-bet abrogates ABC cell production, IgG2a/c expression, and autoantibody production in autoimmune prone strains (39, 43, 71, 72). In the current study, we found that B cell–specific disruption of Hem-1 resulted in increased B220^+^CD11c^+^T-bet^+^ B cells, increased Ag-specific memory and plasma cell formation, increased GC formation, increased IgG2c production in response to immunization with TD Ags, and increased autoantibody production. In addition, purified B220^+^CD21^lo^CD23^+^ B cells from *Hem1^fl/fl^Mb1Cre* mice activated in vitro expressed high mRNA levels of the classical T-bet target gene *Ifng,* whereas *Ifng* transcript levels were either absent or minimally expressed by control B cells. B cell IFN-γ signaling promotes autoimmune GCs and autoantibody production in both mice and humans (73, 74). In 2 independent studies, 50% of the Hem-1 patients with PID were also found to have increased CD19^+^CD38^lo^CD21^–/lo^ ABC-like B cells (21, 22). These results collectively suggest that B cell–specific expression of Hem-1 is important for limiting overproduction of ABC-like T-bet^+^ B cells, and this may contribute to altered B cell behavior and autoimmunity in Hem-1–deficient mice and humans.

T-bet^+^ ABC cell production has been shown to be driven by synergistic BCR, TLR7/9, IL-21, and IFN-γ signaling (37, 49–51). We found that B cell–specific disruption of Hem-1 resulted in increased B cell activation both before and after anti-IgM stimulation, despite lower surface levels of IgM. In addition, the reduction in surface IgM expression was associated with increased IgD expression, characteristics of autoimmune and anergic B cells in both mice and humans (56, 75–78). Replacement of IgM by IgD is thought to be a mechanism for autoreactive B cells to escape deletion upon intense IgM signaling (55, 75). In particular, IgD is less sensitive than IgM in recognizing endogenous antigens, but it is still sufficient to drive GC formation and Ab production. Indeed, we observed that Hem-1–deficient B cells were more efficient at GC B cell, memory B cell, and plasma cell differentiation and IgG2c production in response to immunization with the TD antigens. Our results collectively suggest that B cell–specific expression of Hem-1 is important for modulating B cell fate following BCR-Ag interaction.

In resting B cells, BCRs are maintained on the cell surface in lipid raft bound nanoclusters. Ag binding results in subcortical actin depolymerization and increased BCR diffusion into microclusters, which then recruit and activate key intracellular signaling molecules (79–81). For example, treatment of primary B cells with actin-disrupting agents, or an Arp2/3 inhibitor, are sufficient to increase BCR diffusion and induce Ag-independent, BCR- and CD19-dependent Ca^2+^ signaling, and Erk and AKT phosphorylation equivalent to BCR crosslinking (79, 81–83). Here, we found that B cell–specific disruption of Hem-1 resulted in increased tonic and BCR dependent Ca^2+^ signaling, increased p-ERK, and increased p-S6R signaling, which correlated with reduced IgM and increased IgD expression, changes associated with excessive BCR signaling and autoreactive B cells. We speculate this is due, in part, to disrupted actin polymerization resulting in increased BCR diffusion, clustering, and B cell activation. Interestingly, a recent study found that T cell–specific disruption of WAVE2 resulted in increased T cell activation characterized by increased CD69, increased IFN-γ, and IL-4 expressing cells, increased T-bet^+^CD4^+^ T cells, and increased mTOR activation (84). These results suggest that some of the molecular and cellular functions of Hem-1 and the WRC may be conserved between T and B cells.

In addition to effects on B cells, tissue-wide disruption of Hem-1 in mice and humans has been shown to alter the production and/or behavior of T cells (20, 22, 23), invariant NKT cells (20, 21), neutrophils (20, 23), and macrophages (23, 25, 59), and myeloid cell–specific disruption of murine Hem-1 inhibits the development and alters the functions of alveolar macrophages (25). Our results here collectively provide the first description to our knowledge of how loss of Hem-1 expression specifically in B cells may contribute to severe PID and autoimmunity, and they suggest that Hem-1 and the WRC have critical roles in influencing how B cells respond following a BCR encounter with pathogens and self-derived antigens.

## Methods

Supplemental Methods are available online with this article.

### Mice.

*Nckap1l* floxed mice (*Hem1^fl/fl^*) (25) were bred to *Mb1Cre* mice expressing the Cre recombinase under the control of the *Mb1* promoter (26) to generate mice with B cell–specific deletion of Hem-1. Constitutive *Hem1^–/–^* mice were generated as previously described (25). *Hem1^fl/fl^Mb1Cre* mice were screened and maintained by genomic PCR analysis as described for *Mb1Cre* (26), and following amplification with *Hem1^fl/fl^* forward and reverse oligonucleotides (25). Mice were housed under specific pathogen–free conditions. *Hem1^fl/fl^Mb1Cre* mice were analyzed between generations 3–6 backcrossed on C57BL/6J background. No phenotypic differences were noted between male and female *Hem1^fl/fl^Mb1Cre* mice; thus, both sexes were used equally. The majority of studies were performed on mice ages 8–20 weeks, with the exception of the autoantibody array, which were performed between the ages of 38 and 44 weeks. Littermate controls were utilized whenever possible and in the vast majority of experiments. Experimental controls, denoted WT, included both *Hem1^+/+^* and *Hem1^fl/fl^* mice.

### In vivo migration assay.

B lymphocytes were purified from *Hem1^fl/fl^Mb1Cre* and WT splenocytes by positive selection using CD45R (B220) microbeads (Miltenyi Biotec) according to the manufacturer’s instructions. WT and *Hem1^fl/fl^Mb1Cre* B lymphocytes were stained using CFDA/SE (Invitrogen) and CTV (Invitrogen), respectively. Dye-labeled cells were counted and mixed at a 1:1 ratio prior to i.v. injection. C57BL/6J recipient mice were injected with 1 × 10^6^ dye-labeled B lymphocytes via tail vein injection. Twenty-four hours after injections, spleens, femurs, inguinal LNs, iliac LNs, submandibular LNs, and MLNs were harvested. Cells were isolated, and the transferred cells were tracked using flow cytometry.

### In vitro migration.

Total splenocytes were isolated from *Hem1^fl/fl^Mb1Cre* and WT mice. The lower chambers of transwell plates (5 μm pore, Costar) were loaded with media (RPMI 1640, 2 mM glutamine, and 0.5% BSA or FBS) and incubated at 37°C for 1 hour. CXCL12 (0.2 μg/mL) or CXCL13 (1 μg/mL) was added to the lower chamber prior to the addition of 5 × 10^5^ cells/well into the upper chamber. After 3 hours, the cells were collected from the top and bottom of the chambers and analyzed by flow cytometry. The percentages of migrating B cells were calculated by dividing the absolute number of cells recovered in the lower well divided by the sum of the absolute number of cells recovered in the upper and lower chambers.

### TD and TI immune responses.

Mice between 6 and 10 weeks of age were immunized with 100 μg/mouse NP-KLH (Biosearch Technologies) emulsified in Imject Alum Adjuvant (Thermo Fisher Scientific) or 50 μg/mL NP-Ficoll (50 μg/mL) i.p. For NP-KLH immunization, sera were obtained prior to immunization and then weekly for up to 4 weeks following immunization. Eight weeks after immunization, mice were boosted with 10 μg/mL NP-KLH i.p. For NP-Ficoll immunization, sera were obtained prior to immunization and 6 days after immunization. For sRBC responses, mice were injected with 1 × 10^6^ sRBCs i.p. Eight days after immunization, BM, spleen, and MLN were harvested and analyzed by flow cytometry. For VLP responses, mice were immunized with 10 μg VLP/mouse. Fourteen days after immunization, spleen and MLN were harvested and analyzed by flow cytometry (85).

### Bacteria and pneumococcal infection model.

Frozen *Spn* serotype 2 Strain D39 (gift from Jason Rosch, St. Jude Children’s Research Hospital, Memphis, Tennessee, USA) (1 × 10^8^ cfu) were heat-killed by incubation in a water bath at 60°C duration for 1 hour. Bacterial viability was confirmed by plating 50 μL of the bacterial stock on blood agar plates overnight. The heat-killed bacteria were washed and resuspended in PBS. *Hem1^fl/fl^Mb1Cre* and WT mice were anesthetized with isoflurane and received *Spn* (1 × 10^7^ cfu) via retro-orbital or i.p. injection. Sera were collected from mice prior to and 5 days after HKSP administration to measure Ab titers. For pneumococcal infections, mice were immunized with HKSP (1 × 10^7^ cfu) either i.p. or i.v. Three days later, mice were challenged with *Spn* D39 (1 × 10^7^ cfu) at a lethal dose via i.n. administration. Mice were monitored and weighed daily for 10 days and were euthanized when respiratory distress was observed or when body weight loss exceeded 20% from the day of infection.

### ELISA.

NUNC Maxisorb plates were coated with PC BSA (50 μg/mL) (Biosearch Technologies), NP BSA_2_, or NP BSA_30_ (10 μg/mL) (Biosearch Technologies) and incubated overnight at 4°C. Sera from immunized mice were serially diluted and incubated overnight at 4°C. Sera from unimmunized mice were used as background controls. Ab titers were measured using a spectrophotometer at an OD of 405 nm using horseradish peroxidase–conjugated isotype-specific Abs (Southern Biotech). Baff ELISAs were performed according to manufacturer’s instructions (R&D Systems).

### IgM internalization assay.

Splenocytes (2 × 10^6^ cells/well) were plated and incubated with biotinylated anti-IgM Ab in cell culture media on ice for 30 minutes. Cells were then transferred to 37°C, and IgM internalization was tracked and measured for 0, 2, 5, 10, 15 and 20 minutes by flow cytometry. The presence of cell-surface IgM was measured after staining with streptavidin-conjugated PE in combination with fluorescent conjugated B220.

### B lymphocyte activation.

Splenocytes (2 × 10^6^ cells/well) were stimulated with 10 μg/mL anti-IgM (31178, Invitrogen) or 10 μg/mL anti-IgD Ab (2057001, Invitrogen) for 4, 14, and/or 24 hours. Splenocytes were stained with fluorescent conjugated anti-CD25 (PC61.5, Tonbo Biosciences), anti-CD69 (H1.2F3, BioLegend), and anti-B220 (RA3.6B2, BioLegend) or anti-B220 (RA3-6B2, BioLegend), anti-CD93 (AA4.1 BioLegend), anti-CD21 (7E9, BioLegend), and anti-CD23 (B3B4, BioLegend), and they were analyzed by flow cytometry.

### Flow cytometry.

BM, splenocytes, and LN cells were stained with fluorescent conjugated Abs specific for CD4 (GK1.5, Tonbo Biosciences), CXCR5 (L138D7, BioLegend), PD1 (29F.1A12, BioLegend), CD38 (90, BioLegend), GL7 (GL7, BioLegend), B220 (RA3-6B2, BioLegend), CD93 (AA4.1, BioLegend), CD19 (1D3, Tonbo Biosciences), CD21 (7E9, BioLegend), CD23 (BEB4, BioLegend), CD24 (M1/69, BioLegend), CD62L (MEL-14, Tonbo Biosciences), CD25 (7D4, Tonbo Biosciences), CD43 (S7, BD Pharmigen), IgD (11-26C.2A, BioLegend), and IgM (DS-1, BD Pharmigen); Igκ (1050-02; Southern Biotech); T-bet (EBIO4B10, eBiosciences) and Bcl6 (K112-91, eBioscience); and BrdU (BU20A, BioLegend). Other reagents include Caspase3/7 (C10427, Invitrogen), ghost dye (13-0865-T100, Tonbo Biosciences), and NP-PE (N-5070-1; Biosearch Technologies). Cells were analyzed by flow cytometry as previously described (86). VLP Ab was a gift from Shaun Jackson. Intracellular Bcl6 staining to identify Tfh cells was performed as previously described (87). Intracellular staining for T-bet was performed as previously described (23).

### Protein array profiling analysis.

Mouse sera were collected, aliquoted, and stored at –80ºC. Autoantigen microarrays were manufactured in the Microarray and Immune Phenotyping Core Facility of University of Texas Southwestern Medical Center. A selection of 120 autoantigens was made based on published literature, prior known autoantibodies in various immune-related diseases (cancer, allergic disease, etc.). Two internal control proteins (mouse IgG, anti–mouse IgG), each with 4 different concentrations (100 μg/mL, 50 μg/mL, 25 μg/mL, 12.5 μg/mL) were also imprinted on the arrays as positive and normalization controls. Mouse serum samples were first treated with DNase I to remove free DNA and then applied onto autoantigen arrays at a 1:50 dilution. Autoantibodies binding to the antigens on the array was detected with Cy3-labeled anti–mouse IgG (Jackson ImmunoResearch, AB_2338680) and Cy5-labeled anti–mouse IgM (Jackson ImmunoResearch, AB_2338712), and the array slides were scanned with a Genepix 4400A scanner with laser wavelengths 532 nm for Cy3 and 635 nm for Cy5 to generate TIFF images. Genepix Pro 7.0 software was used to analyze the image and generate GPR files (Molecular Devices). Net fluorescent intensity (NFI) of each antigen was generated by subtracting the local background and negative control (PBS) signal. Signal/noise ratio (SNR = [foreground median – background median]/SD [background]) was generated for each antigen. SNR was used as a quantitative measure of the ability to resolve true signal from background noise. A higher SNR indicates higher signal over background noise. NFI was normalized by a robust linear model using positive controls with different dilutions (88). To avoid outliers in either NFI or SNR, autoantibody scores were calculated using log_2_ (NFI × SNR) + 1. Heatmaps were generated by GENESIS software (Thallinger laboratory, Institute of Bioinformatics, Graz University of Technology, Graz, Austria) on the basis of normalized signal intensity (89).

### Statistics.

Data were analyzed using the Student’s 2-tailed unpaired *t* test with equal variance using GraphPad Prism 6. *P* < 0.05 was considered significant. For multiple comparisons, 2-way ANOVA with Tukey’s multiple-comparison test was utilized. For Kaplan Meier analyses, differences were tested using Gehan-Breslow-Wilcoxon test by GraphPad Prism.

### Study approval.

All studies involving animals were approved by the University of Washington Animal Care and Use Committee.

## Author contributions

AA, HP, SWJ, and BMI designed the experiments and wrote the manuscript; AA, HP, JTT, KKB, NS, JDW, CM, and AC collected and analyzed data; CZ, and QZL generated and analyzed autoantibody array data; and HDL analyzed histology sections.

## Supplementary Material

Supplemental data

## Version 1 05/09/2022

Electronic publication
