# Supplementary material for: Hem-1 regulates protective humoral immunity and limits autoantibody production in a B cell–specific manner
Source: JCI Insight. 2022 May 9;7(9):e153597. doi: 10.1172/jci.insight.153597 (PMC9090261; doi:10.1172/jci.insight.153597)
Supplement: Supplemental data [file jciinsight-7-153597-s101.pdf]

Supplementary Materials

Supplemental Materials and methods

In vivo tracking sinusoidal B cell populations

In vivo assessment of B cells located in sinusoids versus parenchyma was performed by injecting *Hem1*$^{fl/fl}$*Mb1-Cre* and WT mice with 0.1 µg/mouse PE-labeled anti-CD19 Ab (clone 1D3, BD Biosciences) via retro-orbital injection while mice were under anesthesia. Mice were induced with 5% isoflurane in an induction chamber followed by maintenance on 0.5-2% isoflurane via a nose cone. Two min after injection, mice were euthanized immediately and BM and spleen were collected. Cells were analyzed with flow cytometry

Bacteria and pneumococcal infection model

Frozen *Streptococcus pneumoniae* serotype 2 Strain D39 (10^8cfu) were heat-killed by incubation in a water bath (60°C) duration one hour. Bacterial viability was confirmed by plating 50 uL of the bacterial stock on blood agar plates overnight. The HKSP were washed and resuspended in PBS. *Hem1*$^{fl/fl}$*Mb1Cre, Hem1*$^{-/-}$*,* and WT mice were anesthetized within an isoflurane chamber and received HKSP *(*10^7 cfu) via retro- orbital or i.p. injection. Sera was collected from mice prior to and 5 days after HKSP administration to measure Ab titers. For pneumococcal infection, mice were immunized with HKSP (10^7 cfu) either i.p. or i.v. Three days later, mice were challenged with live *S. pneumoniae* (10^7 cfu, D39 strain) via intranasal route while under anesthesia. Mice were monitored and weighed daily for 10 days and were euthanized when respiratory distress or body weight loss (≥20%) was observed.

Calcium influx assay

Intracellular Ca^{2+} influx was measured with Fluo-4 Direct™ Calcium Assay Kits (ThermoFisher) according to the manufacturer's protocol. Briefly, splenic B cells ($5x10^6$ cells) were washed with Ca^{2+}-free PBS and were added with 50 µL of the 2X Fluo-4 Direct™ calcium reagent loading solution per well of a 96-well plate containing 50 µL of culture medium (RPMI1640 plus 10% FBS) per well. Cells were incubated at 37°C for 30 minutes. Calcium influx was detected with LSRII flow cytometer (Becton Dickinson, Franklin Lakes, NJ) on FITC channel as a function of time. After collecting a baseline measurement for 1 minute, samples were stimulated by adding 10 µg/ml anti-µ (F(ab′)2 fragment) or anti-IgD. Change of fluorescence were plotted as a function of time. Ionomycin (final conc, 1ug/ml) stimulation was used for positive control. Data were analyzed using FlowJo 4 software (10.7.1). Baseline, peak anti-IgM, peak ionomycin Fluo-4 ratios were gated as described in Supplemental Figure 9B.

Measurements of autoantibodies

Specific autoAb ELISAs were performed as previously described(90). Briefly, plates were coated with goat anti-mouse IgM, IgG, or IgG2c Abs (1:500 dilution; Southern Biotech), followed by blocking with 1% BSA prior to incubation with diluted serums. Specific Abs were detected using goat anti-mouse IgM, IgG, or IgG2c/HRP (1:2000 dilution; Southern Biotech), and perioxidate reactions were developed using OptEIA TMB substrate (BD Biosciences). Absorbance was determined at 450 nm. For autoantigen microarrays, sera

were sent to UT Southwestern Medical Center Genomics and Microarray core facility. Sample were hybridized to the Autoantigen Microarray Super Panel with 128 antigens.

Histology

Tissues were fixed in formalin, and embedded, sectioned and stained with H&E or PAS at Histology Consultation Services (Everson, Wa). The number of GCs were evaluated in a blinded fashion and normalized for the area of the histologic section that was evaluated using NIS-Elements (Nikon). The area of the individual GCs were measured and averaged per mouse (n=8 WT and n=6 *Hem1*$^{fl/fl}$*Mb1Cre*).

Immunoblot analysis

B lymphocytes were purified from *Hem1*$^{fl/fl}$*Mb1-Cre* and WT splenocytes by positive selection using CD45R (B220) microbeads (Miltenyi Biotec) according to the manufacturer's instructions. Immunoblot analyses were performed as previously described (91) using rabbit polyclonal antibodies or mouse monoclonal antibodies specific for βActin, Akt phospho-Ser473, p-Ser240/244 S6 Ribosomal Protein, p-Erk (Cell Signaling), followed by α-rabbit IgG HRP (Promega).

Quantitative RT-PCR

Splenocytes were isolated from female *Hem1*$^{fl/fl}$*Mb1Cre* and WT control mice. Splenocytes were stained with fluorescently labeled antibodies against B220,CD21,CD23 and B220$^+$CD23$^+$CD21^{lo} cells were FACs sorted on a BDFACSAria III. 1.5 x 10^6 purified cells were stimulated with 10 μg/ml anti-IgM and 20IU/ml IFNg for 12 hrs in B cell growth media (RPMI + glutamine, 10%FCS). Total RNA was prepared using RNAplus minikit

(Qiagen #74134). cDNAs were prepared (and validated) from 0.5 ng total RNA using SMARTseqV4 Ultra low Input RNA kit for sequencing (Takara) according to manufacturer’s instructions by the Genomics Core, Benaroya Research Institute at Virginia Mason, Seattle WA.. Quantitative real-time PCR was performed for both *Ifng* and *gapdh* (endogenous control) using the Quantinova SYBR Green RT-PCR kit (Qiagen, 208152) on a Strategene Mx3005 qPCR System. A total of two microliters (approximately 20-100ng) of cDNA was loaded into each reaction. Thermal cycling conditions followed manufacturer's instructions. *Infg* forward primer (GGCCATCAGCAACAACATAAGCGT) *Ifng* reverse (TGGGTTGTTGACCTCAAACTTGGC); *Gapdh* forward primer (GTTGTCTCCTGCGACTTCA); *Gapdh* reverse primer (GGTGGTCCAGGGTTTCTTA).

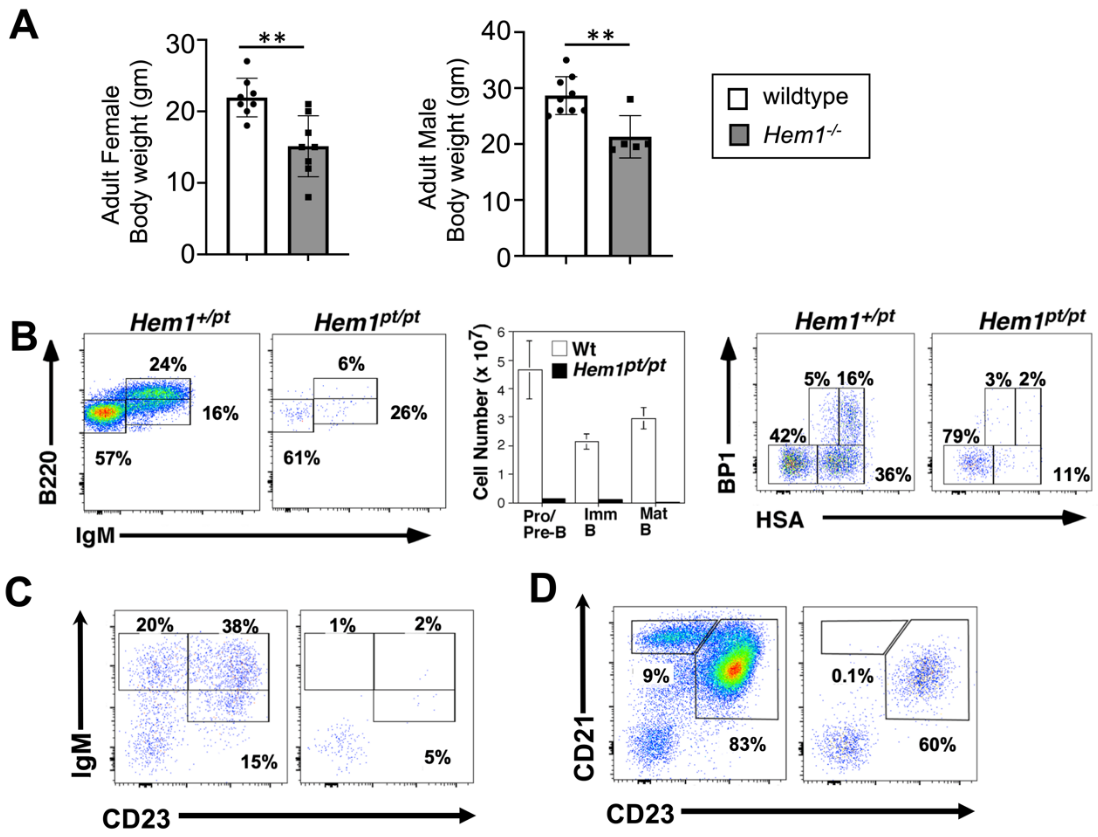

Supplemental Figure 1. Hem-1 null mice are smaller in size and exhibit impaired central and peripheral B cell development.
(A) Adult body weight of female (*left,* 58-134 days age) and male (*right,* 58-155 days age) WT and *Hem1*$^{-/-}$ mice. (B-D) BM and splenocytes were harvested from 8 -12-wk-old *Hem1*$^{pt/pt}$ (23) and WT control mice. Shown are representative flow cytometric histograms of (B) BM B cell populations demonstrating a reduction of B cells beginning at Hardy Fraction B (B220^{+}CD43^{+}HSA^{mid}BP1^{neg}IgM^{-}); and (C,D) splenocytes demonstrating a reduction of transitional and mature populations. Data are representative of multiple (>10) independent experiments.

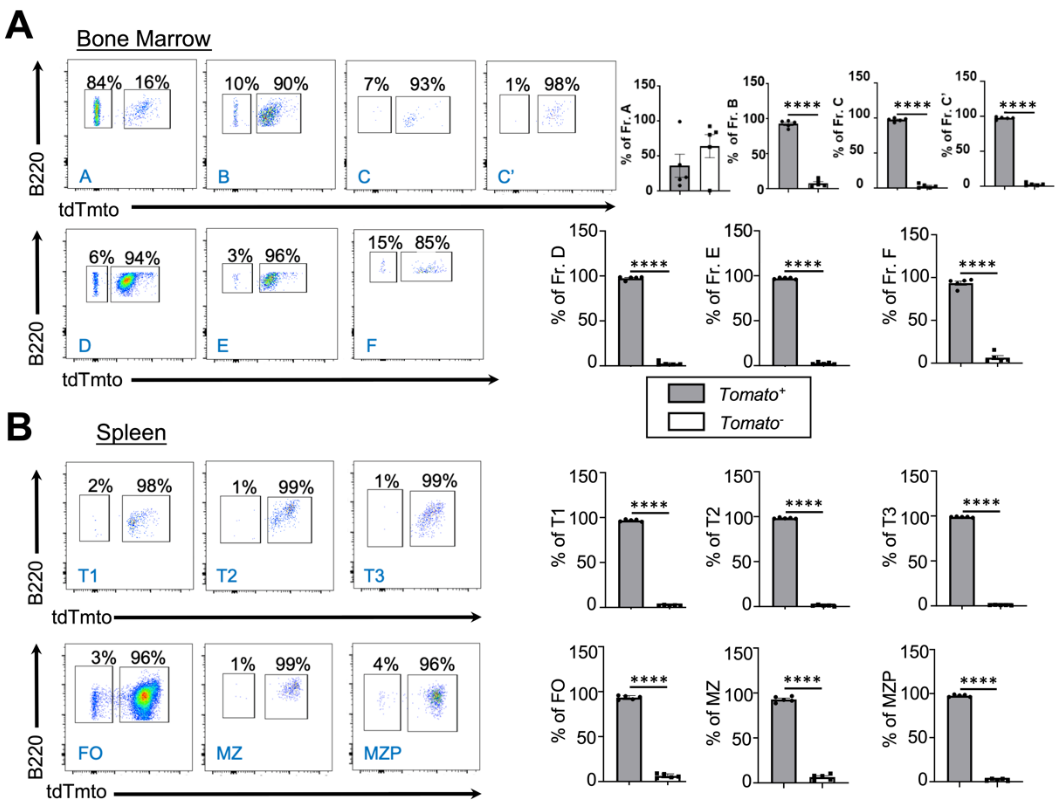

Supplemental Figure 2. The reporter TdTomato demonstrates strong Cre recombinase activity in *Hem1^fl/fl^Mb1Cre* mice throughout B cell maturation.
BM and splenocytes were harvested from *Hem1^fl/fl^Mb1Cre TdTmto* and WT control mice followed by flow cytometric analyses. (A) Expression of Tomato RFP in B cell progenitors in BM reveals Cre expression beginning in the Hardy Fraction B stage. Shown are representative dot-plot histograms (*left*) and bar graphs (*right*) showing expression of tdTomato in gated Hardy Fractions A ($B220^{+}CD43^{+}HSA^{lo}BP1^{neg}$), B ($B220^{+}CD43^{+}HSA^{mid}BP1^{neg}$), C ($B220^{+}CD43^{+}HSA^{hi}BP1^{+}$), C' ($B220^{+}CD43^{+}HSA^{hi}BP1^{-}$), D ($B220^{lo}CD43^{-}IgM^{-}$), E ($B220^{lo}CD43^{-}IgM^{+}$), F ($B220^{hi}CD43^{-}IgM^{+}$). (B) Expression of tdTmto in splenic B cells derived from *Hem1^fl/fl^Mb1Cre TdTmto* and *Hem1^fl/fl^ Mb1*Cre control mice reveals strong Cre expression throughout peripheral B cell development. Shown are representative dot plot histograms (*left*) and bar graphs (*right*) showing tdTomato expression in gated T0 ($B220^{+}CD93^{+}IgM^{-}CD23^{-}IgD^{-}$), T1($B220^{+}CD93^{+}IgM^{+}CD23^{-}$), T2($B220^{+}CD93^{+}IgM^{+}CD23^{+}$), T3($B220^{+}CD93^{+}IgM^{lo}CD23^{+}$), MZ($B220^{+}CD93^{-}CD21^{+}CD23^{lo}$), and FO ($B220^{+}CD93^{-}CD21^{lo}CD23^{hi}$) B cells. Data are presented as the percentage of tdTmto+ or tdTmto- in each population. Each dot represents an individual mouse. Data are representative of 2 independent experiments (n=5 mice per group). Data represent the mean ± SEM and were analyzed via paired Student's t-test *p<0.05, **p<0.01, *** p<0.001.

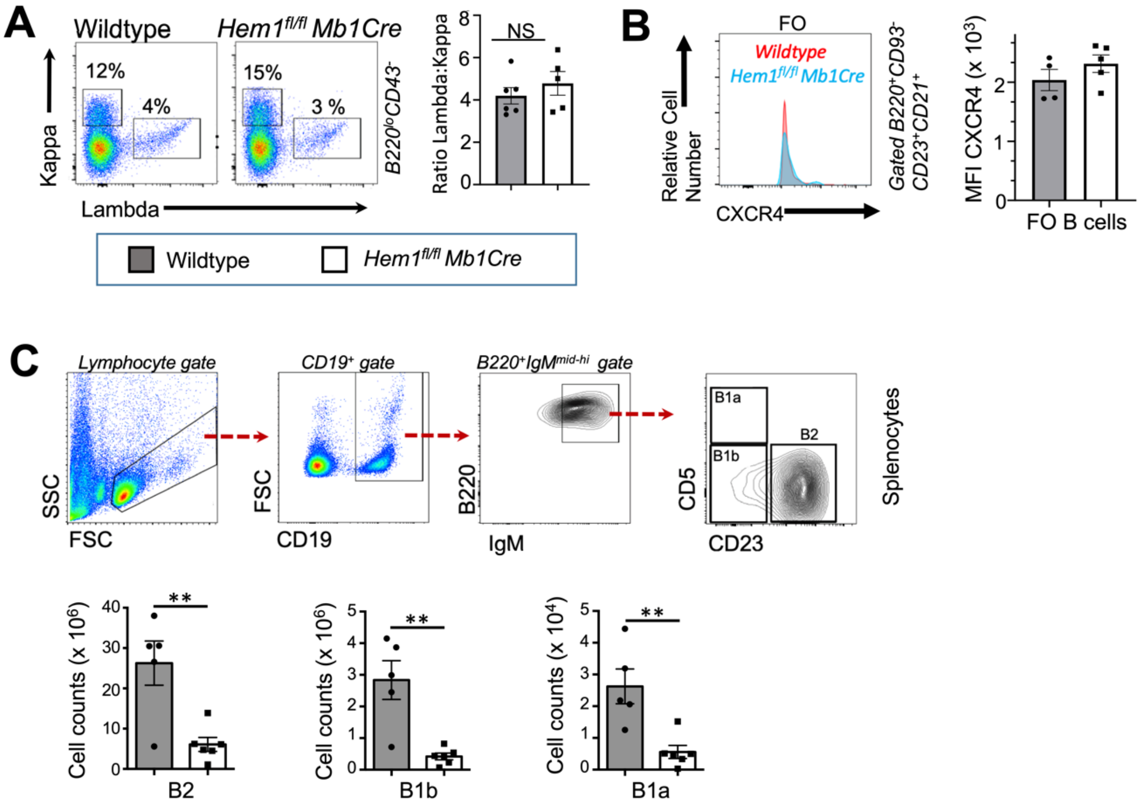

Supplemental Figure 3. Normal ratio of IgKappa, IgLambda, and CXCR4 expression, and reduced B1 B cell production following B cell specific disruption of Hem1.

Splenocytes were harvested from 6-12-wk-old WT and *Hem1$^{fl/fl}$Mb1-Cre* mice, followed by flow cytometric analyses. (A)Shown are representative dot-plot histograms (*left*) and a bar graph (*right*) showing equivalent expression of Ig λ and κ light chains on gated B220^{lo}CD43^{-} immature B cells. (B) Representative flow cytometric single parameter histograms showing equivalent CXCR4 expression on B220^{hi}CD93^{-}CD21^{lo}CD23^{+} cells from WT and *Hem1$^{fl/fl}$Mb1Cre* mice. Each data point is representative of an individual mouse and the data are representative of $\geq$ 2 separate experiments. (C) Shown are representative dot-plot histograms (*top*) and a bar graph (*bottom*) showing the numbers of B2, B1b, B1a, and B2 B cells. N=4-6 mice per group. Data represent mean ± SEM and analyzed via unpaired Student's t-test. *p<0.05, **p<0.01, *** p<0.001

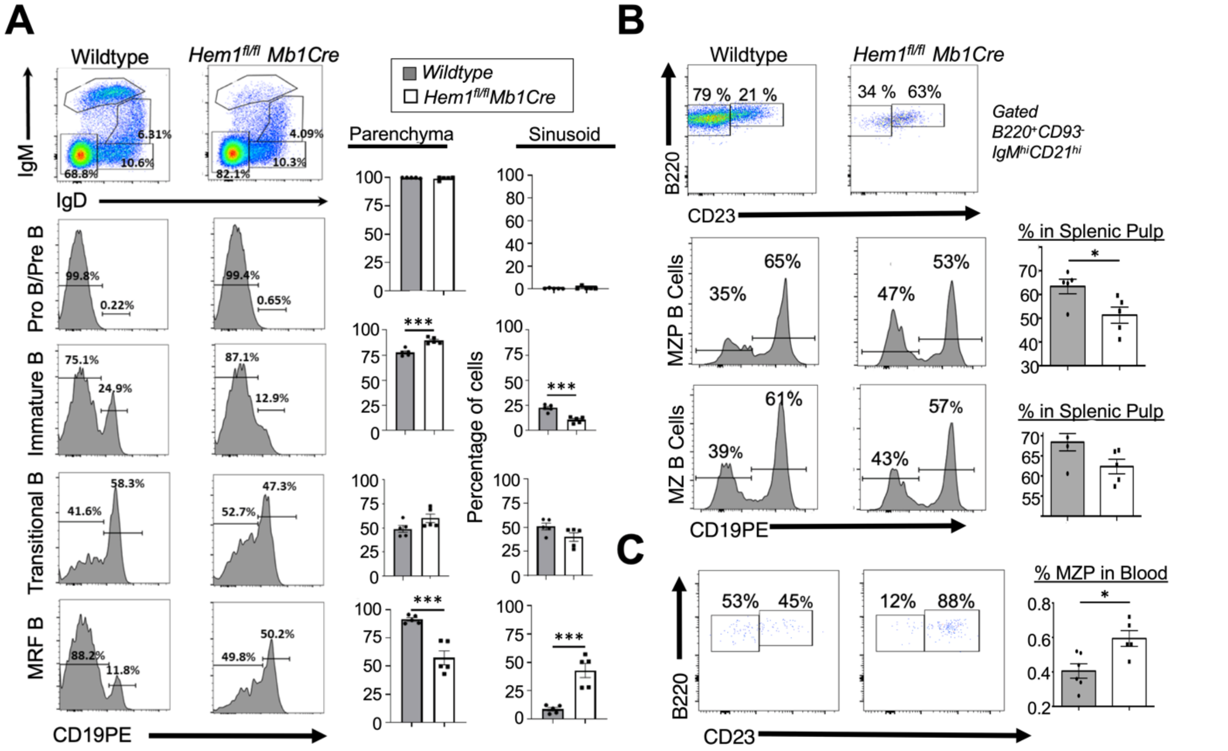

Supplemental Figure 4. In vivo labeling reveals increased representation of mature recirculating follicular B cells and marginal zone precursor B cells in the splenic blood-filled sinusoids of *Hem1$^{fl/fl}$Mb1-Cre* mice.

(A) WT and *Hem1$^{fl/fl}$Mb1Cre* mice were anesthetized and injected with 0.1 μg/mL PE-labeled anti-CD19 Ab intravenously, followed by euthanasia 2 min post-injection. Shown are representative flow cytometric histograms (*left*) and bar graphs (*right*) demonstrating the representation of pre-B/pro-B (IgM^-IgD^-), immature (IgM^+IgD^-), transitional ($IgM^+IgD^{int}CD93^+$), and mature follicular (IgD^+IgM^+) B cell populations in the BM parenchyma ($CD19PE^-$) versus blood-filled sinusoids ($CD19PE^+$). Each data point represents an individual mouse and the data are representative of two independent experiments (n=5 and n=4). (B) Mice were anesthetized and injected with 1 μg/mL PE-labeled anti-CD19 Ab i.v., followed by euthanasia 5 min post-injection. Shown are representative flow cytometric histograms and bar graphs demonstrating the representation of MZ ($B220^+CD93^-IgM^+CD21^{hi}CD23^-$) and MZ precursor ($B220^+CD93^-IgM^+CD21^{hi}CD23^+$) B cells that were in the splenic parenchyma ($CD19PE^-$) versus B cells labeled in the splenic blood filled sinusoids ($CD19PE^+$). Each data point represents an individual mouse and the data are from one experiment for MZ and MZP B cells in spleen (n=6 and n=5), and MZP in the blood (n=6 and n=6). Data represent mean ± SEM and analyzed via unpaired Student's t-test. *p<0.05, **p<0.01, *** p<0.001

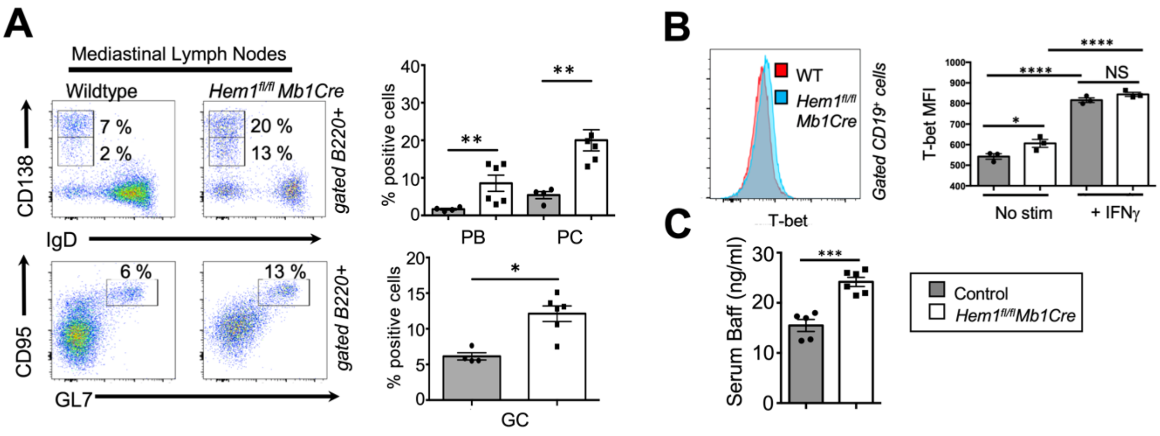

Supplemental Figure 5. Conditional disruption of Hem1 results in increased T-bet positive B cells in response to IFNgamma and increased representation of plasmablasts, plasmacells, and GC B cells in response to influenza virus challenge. (A) WT (n=5) and *Hem1$^{fl/fl}$Mb1Cre* (n=4) mice were infected with the mouse adapted influenza virus PR/8 strain. 10 days post-infection, mediastinal lymph nodes were collected and analyzed for germinal center (B220^{+}CD95^{+}GL7^{+}), plasmablast (B220^{+}IgD^{int}CD138^{int}) and plasma cell (B220^{+}IgD^{int}CD138^{hi}) representation by flow cytometry. Shown are representative flow cytometric histograms (*left*) and bar graphs (*right*) depicting the representation of plasma cells (PC), plasmablasts (PB) and germinal center (GC) B cells. Each dot represents individual mice. (B) B cell specific disruption of Hem-1 results in increased expression of T-bet in B cells. Splenocytes were isolated from WT and *Hem1$^{fl/fl}$Mb1Cre* mice, followed by in vitro stimulation with or without IFNγ for 3 days. 10^5 cells were stained with fluorescent-conjugated α-CD19 and IC α-T-bet and analyzed by flow cytometry. Shown are representative single parameter histograms of T-bet expression *(left)* and a bar graphs showing MFI of T-bet expression *(right)* on gated CD19^{+} B cells. N= 3 mice per group. (C) Sera were collected from 8-9 week old *Hem1$^{fl/fl}$ Mb1Cre* and littermate control mice. Shown are serum Baff levels (ng/ml) determined by ELISA. (n=5-6 per group). Representative of one assay. Each dot represents individual mice. Data represent mean ± SEM and analyzed via unpaired Student's t-test. *p<0.05, **p<0.01, *** p<0.001

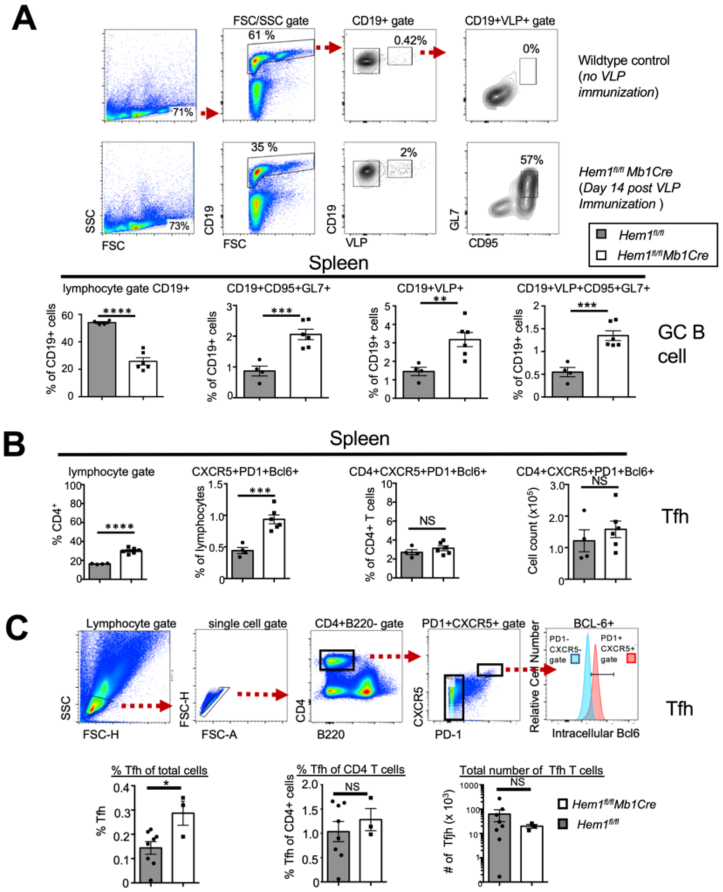

Supplemental Figure 6. B cell specific disruption of Hem1 results in increased representation of antigen-specific GC B cells following immunization with virus-like particles . *Hem1$^{fl/fl}$Mb1Cre* and *Hem1$^{fl/fl}$* littermate control mice were immunized with 10 μg VLP in 250 μl sterile PBS. Splenocytes and MLN were harvested and stained with fluorescent conjugated antibodies against CD19, VLP, CD95, GL6 or CD4,B220, CXCR5, PD1 and intracellular Bcl6. Shown are (A) (*top*) representative multiparameter histograms and gating strategies from unimmunized WT control and immunized *Hem1$^{fl/fl}$Mb1*Cre mice 14 days post-immunization; (*bottom*) bar graphs showing the representation and total numbers of CD19$^+$ B cells, CD19$^+$CD95$^+$GL7$^+$ total GC B cells, CD19$^+$VLP$^+$ B cells, and CD19$^+$VLP$^+$CD95$^+$GL7$^+$ VLP-specific GC B cells. (B) Shown are bar graphs depicting the percentage of B220$^-$CD4$^+$ T cells, B220$^-$CD4$^+$CXCR5$^+$PD1$^+$Bcl6$^+$ Tfh cells relative total lymphocytes, B220$^-$CD4$^+$CXCR5$^+$PD1$^+$Bcl6$^+$ Tfh cells relative to CD4+ cells, and the total number of B220$^-$CD4$^+$CXCR5$^+$PD1$^+$Bcl6$^+$ Tfh cells. (C) *Hem1$^{fl/fl}$Mb1Cre* and *Hem1$^{fl/fl}$* littermate control mice were immunized with 1 x 10^6 sRBCs i.p. in 250 μl sterile PBS. Spleens were harvested 8 days post-immunization and splenocytes stained with fluorescent conjugated antibodies. Shown are (top) dot-plot histograms showing gating strategies for defining Tfh cells, and (bottom) bar graphs depicting the percentage of B220$^-$CD4$^+$CXCR5$^+$PD1$^+$Bcl6$^+$ Tfh cells relative total lymphocytes, B220$^-$CD4$^+$CXCR5$^+$PD1$^+$Bcl6$^+$ Tfh cells relative to CD4+ cells, and the total number of B220$^-$CD4$^+$CXCR5$^+$PD1$^+$Bcl6$^+$ Tfh cells. n=4-8 mice per group. **<0.01, ***<0.005, ****<0.001

A

Autoantigen	$Hem1^{fl/fl}$ *Mb1Cre* mean IgG	Cont Mean IgG	p-value	cKO vs cont
Factor D	13.5	11.8	0.001	up
Core Histone	11.4	9.2	0.002	up
complement C1q	20.6	18.3	0.003	up
SmD3	13.3	11.4	0.003	up
Aggrecan	11.2	10.0	0.003	up
Histone H2A	20.2	18.3	0.005	up
Heparan Sulphate	7.5	1.8	0.007	up
Histone H3	13.9	12.1	0.007	up
B2-microglobulin	11.0	9.5	0.009	up
Factor B	19.5	18.3	0.009	up
Fibronectin	17.4	15.7	0.012	up
PR3	12.5	10.2	0.014	up
GP2	19.1	17.4	0.015	up
Sm/RNP	16.1	14.9	0.015	up
Heparan sulfate proteoglycan	13.8	12.2	0.018	up
U1-snRNP C	14.4	13.5	0.018	up
complement C3a	15.1	13.8	0.019	up
SmD1	12.0	10.1	0.020	up
Histone H2B	17.7	16.4	0.020	up
SP100	13.9	12.3	0.020	up
U1-snRNP 68/70	17.4	16.5	0.023	up
Laminin	12.9	11.5	0.026	up
Mitochondrial antigen	16.8	15.4	0.028	up
PCNA	19.2	18.2	0.029	up
SARS-CoV-2 Spike S1 RBD	19.2	18.0	0.030	up
Histone H1	15.8	14.8	0.030	up
complement C5	17.3	16.0	0.032	up
Myelin basic protein (MBP)	17.6	16.7	0.034	up
LC1	20.1	18.2	0.042	up
SARS-CoV-2 NCP	11.9	10.8	0.042	up
U1-snRNP B/B'	15.3	14.0	0.042	up

B

Autoantigen	$Hem1^{fl/fl}$ *Mb1Cre* mean IgM	Control Mean IgM	p-value	cKO vs cont
Nucleosome antigen	20.6	0.82	0.019	up
U1-snRNP B/B'	16.1	1.19	0.019	up
SmD1	12.1	1.38	0.022	up
Insulin	12.6	1.40	0.022	up
Myelin basic protein (MBP)	19.4	0.73	0.022	up
Sm	12.9	1.58	0.022	up
complement C7	18.6	1.40	0.027	up
MPO	17.1	1.32	0.029	up
Histone H4	11.7	1.63	0.031	up
Factor D	13.2	1.41	0.031	up
T1F1 gama	14.9	1.35	0.032	up
TPO	18.2	1.28	0.032	up
complement C3a	16.2	0.91	0.033	up
Histone H2B	17.8	1.87	0.033	up
AGTR	16.1	1.55	0.033	up
Elastin	11.8	1.41	0.034	up
Histone H3	13.1	1.49	0.038	up
Collagen IV	13.2	1.23	0.040	up
Ribo Phosphoprotein P2	13.4	1.35	0.044	up
Prothrombin protein	18.7	1.54	0.045	up
Vitronectin	16.1	1.41	0.045	up
TNF-alpha	18.4	1.67	0.045	up
SmD	13.0	0.88	0.046	up
Chondroitin Sulfate C	3.0	0.95	0.046	up
Heparin	3.7	1.37	0.046	up
TTG	19.9	1.67	0.047	up
GP2	19.2	1.74	0.047	up
complement C8	18.8	1.55	0.048	up
Entaktin EDTA	13.0	1.12	0.048	up
Collagen I	8.4	1.55	0.050	up

Supplemental Figure 7. Autoantigen microarray reveals Increase autoantibodies following B cell specific disruption of Hem-1.

Sera were collected from female $Hem1^{fl/fl}$*Mb1Cre* and control mice ages 28-32 and hybridized to an Autoantigen Microarray containing 128 antigens. Shown are charts depicting statistically significant changes in IgM (left) and IgG (right) autoantibody levels in $Hem1^{fl/fl}$*Mb1Cre* sera relative to control sera. Data represent mean antibody score analyzed via unpaired Student's t-test. p-values are shown. N=5 WT and 3 $Hem1^{fl/fl}$*Mb1Cre* mice. Data are representative of a single autoantigen array experiment.

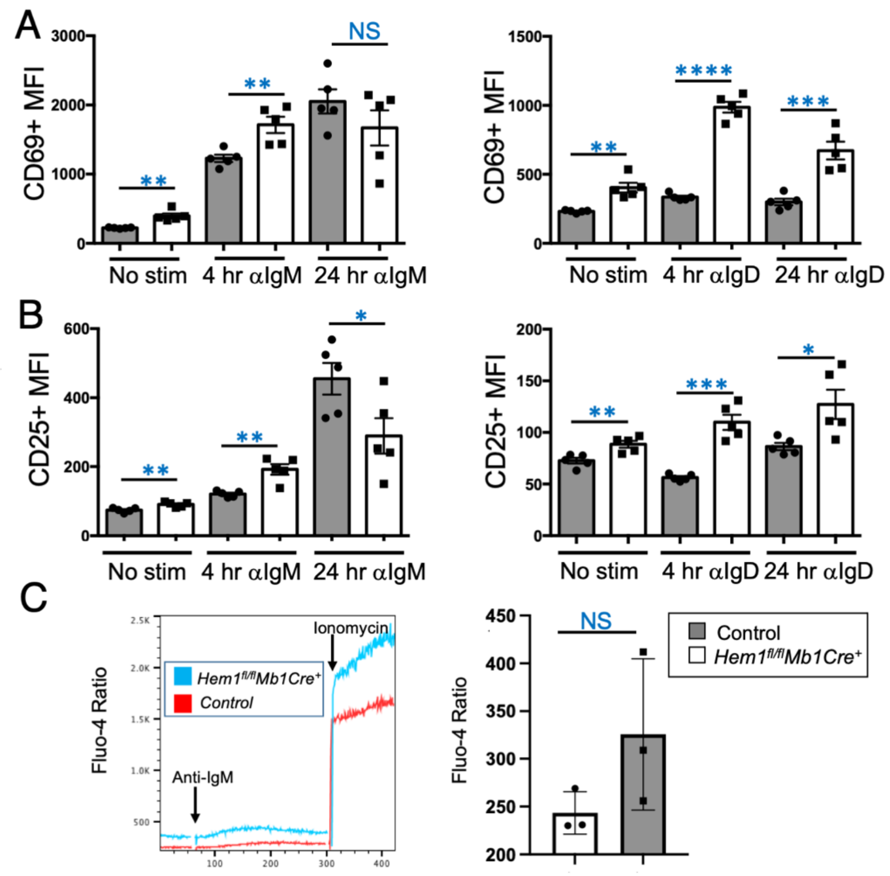

Supplemental Figure 8. B cell specific disruption of Hem-1 results in increased expression of CD25 and CD69 basally and in response to BCR stimulation.
Splenocytes were isolated from WT and *Hem1$^{fl/fl}$Mb1Cre* mice, followed by in vitro stimulation with α-IgM (10μg/ml) or IgD (10μg/ml) for 4hrs or 24hrs. 10^5 cells were stained with fluorescent-conjugated α-B220, α-CD25, and α-CD69 and analyzed by flow cytometry. Shown are bar graphs showing MFI of (A) CD69 or (B) CD25 expression on gated B220$^+$ cells that were unstimulated, IgM stimulated (*left*) or IgD stimulated B cells (*right*). N= 5 mice per group. (C) Total splenic B cells from WT and *Hem1$^{fl/fl}$Mb1Cre* mice were stained with Fluo-4 and stimulated with 10 μg/ml anti-IgM followed by ionomycin to determine BCR induced calcium influx. Data is representative of two experiments (n=3 and n=3). Data represent mean ± SEM and were analyzed via unpaired Student's t-test. *p<0.05, **p<0.01, *** p<0.001, **** p<0.0001

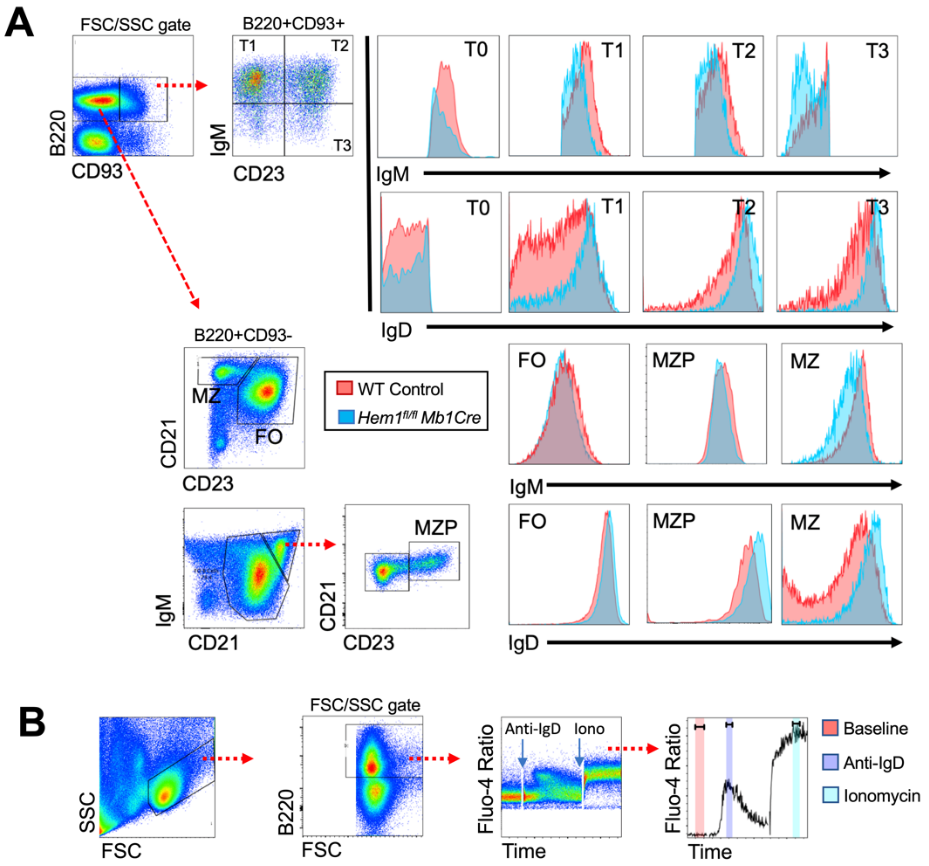

Supplemental Figure 9. B cell specific disruption of Hem1 results in altered expression of IgM, IgD and increased intracellular calcium levels.

Splenocytes were harvested from 12-wk-old WT and *Hem1$^{fl/fl}$Mb1-Cre* mice and stained with fluorescent conjugated antibodies against B220, CD93, IgM, CD23, CD21 followed by flow cytometric analyses. (A) Shown are representative dot-plot and single parameter histograms showing gating strategies and the relative expression of IgM and IgD on T0, T1, T2, T3, FO, MZP, and MZ B cells. (B) Splenocytes were stained with fluorescent conjugated antibodies against B220 and loaded with Fluo-4 dye. Fluo-4 ratios were determined by flow cytometry over a 10 minute period. Anti-IgD 10μg/ml and ionomycin (final concentration 1μg/ml) were added at the indicated timepoints. Shown are representative dot-plot and single parameter flow cytometric histograms and gating strategies used to determine Fluo-4 ratios at baseline, peak anti-IgD, and peak ionomycin stimulation.
